# Mongolian medicine Eerdun-Wurile promotes myocardial regeneration by regulating MVDA in zebrafish

**DOI:** 10.1186/s13619-025-00235-z

**Published:** 2025-06-06

**Authors:** Xianghui Chen, Xiaoting Li, Jiajun Sun, Yufeng Lin, Yuanhao Li, Xuehao Lv, Rui Zhao, Xinyue Gu, Wenxuan Wang, Yabin Xie, Wei Xie, Rengui Bade, Shuyuan Jiang, Xiaolei Liu, Bo Zou, Yannan Bi, Guo Shao, Haihua Bai, Wei Zhu, Xiaoe Jia

**Affiliations:** 1 Department of Basic Medicine and Forensic Medicine, Baotou Medical College, Baotou, Inner Mongolia 014040 China; 2 Inner Mongolia Key Laboratory of Hypoxic Translational Medicine, Baotou Medical College, Baotou, Inner Mongolia 014040 China; 3 Department of Clinical Laboratory, Traditional Chinese Medicine Hospital of Jiamusi, Jiamusi, Heilongjiang 154002 China; 4 School of Public Health, Baotou Medical College, Baotou, Inner Mongolia China; 5 Beijing Key Laboratory of Hypoxic Conditioning Translational Medicine, Xuanwu Hospital, Capital Medical University, Beijing, China; 6 School of Pharmacy, Baotou Medical College, Baotou, Inner Mongolia 014040 China; 7 Center for Translational Medicine and Department of Laboratory Medicine, The Third People’s Hospital of Longgang District, Shenzhen, China; 8 Affiliated Hospital of Inner Mongolia Minzu University, Tongliao, 028000 China

**Keywords:** Cardiomyocytes, Regeneration and repair, MVDA, Proliferation

## Abstract

Myocardial infarction (MI) is a leading cause of death and disability worldwide. The promotion of myocardial regeneration is a promising therapeutic strategy for acute MI. Using a zebrafish ventricular ablation system, we found that the Mongolian traditional medicine Eerdun-Wurile (EW) promotes myocardial regeneration in zebrafish. EW treatment significantly accelerated proliferation of myocardial cells and improved cardiac function. Transcriptome sequencing revealed a significant decrease in mevalonate diphosphate decarboxylase a (*mvda*) expression in the metronidazole-induced ventricular ablation group, whereas *mvda* expression was restored in the EW group. *mvda* knockdown using morpholino oligonucleotides reversed the EW-mediated myocardial regeneration, whereas *mvda* overexpression enhanced the regenerative ability. In conclusion, EW may promote zebrafish myocardial regeneration, accelerate myocardial cell proliferation, and improve cardiac function by upregulating *mvda* expression. Our data partially revealed the molecular mechanism by which EW promotes myocardial regeneration and repair, and provides experimental data and novel insights for advancing MI treatment.

**Supplementary Information:**

The online version contains supplementary material available at 10.1186/s13619-025-00235-z.

## Background

Cardiovascular disease (CVD) is a leading cause of mortality (Thygesen et al. 2012). Approximately 17 million people die of CVDs worldwide, accounting for one-third of all deaths. China has the highest number of CVD-related deaths (Roth et al. 2020). Thus, CVDs pose a significant threat to human health. Myocardial cells undergo necrosis and apoptosis following myocardial infarction (MI). Drug therapy and bypass surgery may relieve symptoms and restore blood supply; however, these methods cannot repair damaged myocardial cells (Garbern & Lee 2022; Giacca 2020). Myocardial regeneration represents a novel therapeutic strategy that can fundamentally restore the heart function (Garbern & Lee 2022; Giacca 2020).

In newborn mammals, cardiomyocytes (CMs) can regenerate following injury. However, this regenerative ability is lost after 7 postnatal days (Porrello et al. 2011, 2013; Sadek & Olson 2020). Many studies have provided evidence for adult mammalian cardiac regeneration; however, the processes involved in the efficient conversion of mammalian CMs into regenerative phenotypes remain largely unknown. Unlike mammals, zebrafish can regenerate CMs following cardiac injury (Gemberling et al. 2013). Therefore, identifying the cellular and molecular mechanisms underlying CM regeneration in zebrafish represents a promising approach to gain insights into cardiac regeneration in mammals.

The Mongolian medicine Eerdun-Wurile (EW) is commonly used for the clinical treatment of central nervous system diseases such as nerve injury, cerebral haemorrhage, cerebral thrombosis, and CVDs. EW is composed of 29 individual medicinal ingredients, including *Terminalia chebula Retz* (fruits), *Carthamus tinctorius* (flowers), *Gardenia jasminoides Ellis* (fruits), *Amomum tsaoko* (fruits), *Glycyrrhiza uralensis Fis* (roots and rhizomes), *Myristica fragrans* (seeds), *Abutilon theophrasti* (seeds), *Melia toosendan Sieb* (fruits), *Cassia obtusifolia* (seeds), *Saussurea costus* (roots), and *Cinnamomum cassia* (bark). A recent study showed that EW improved stroke recovery in a rat middle cerebral artery occlusion model through the regulation of *IGF1*, *IGF2*, and *TGFβ* gene expression. Another study found that EW enhances the polarisation of microglia from the M1 to M2 phenotype, which may contribute to nerve repair and neurogenesis (Gaowa et al. 2018). EW restores postoperative cognitive dysfunction by suppressing the TLR4/NF-κB pathway activation and microglia and activating of the PI3K signalling pathway (Lv et al. 2021; Qiao et al. 2023). Although the therapeutic effects of EW in cardiac diseases have been demonstrated previously, the underlying protective mechanisms remain unclear.

Therefore, we used the zebrafish ventricular ablation system to analyse the effect of the Mongolian medicine EW on promotion of CM repair and examine the underlying mechanisms. We found that EW promoted myocardial regeneration, accelerated myocardial cell proliferation, and improved cardiac function in zebrafish by upregulating mevalonate diphosphate decarboxylase a (MVDA) (encoded by *mvda*).

## Results

### Successful construction of the myocardial injury regeneration model

**Fig. 1:**
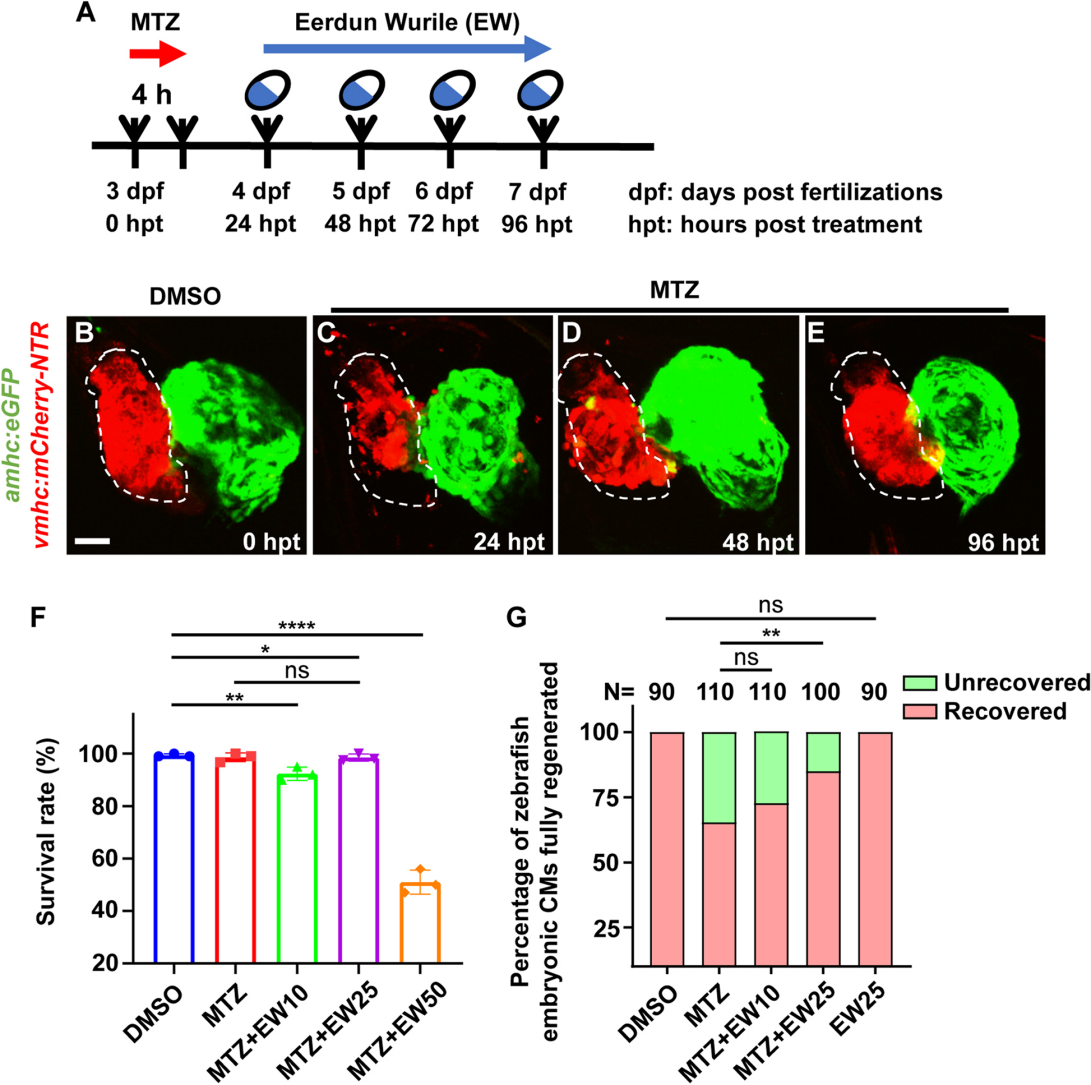
Successful construction of a myocardial injury-repair model. Schematic of the experimental timeline (**A**). Representative images of ventricular morphology of zebrafish in the control (**B**) and ventricular ablation (**C**-**E**) groups after metronidazole (MTZ) treatment. Survival rates of zebrafish after treatment with different concentrations of EW (**F**). Statistical analysis of data for complete myocardial regeneration at different Eerdun-Wurile (EW) concentrations (**G**). *, *P* < 0.05; **, *P* < 0.01; ****, *P* < 0.0001. Scale bar, 20 μm

To study the effect of EW on cardiac regeneration, we used a genetic cardiac ventricle-specific nitroreductase (NTR)-mediated ablation system in zebrafish Tg (*vmhc:*mCherry-NTR). The transgenic fish line Tg (*vmhc:*mCherry-NTR) expresses the red fluorescent protein mCherry fused with NTR under the control of the ventricular myosin heavy chain (*vmhc*) promoter. The transgenic zebrafish embryos (3 dpf) were treated with 5 mM Metronidazole (MTZ) for 4 h (Fig. 1A). In this model, the interaction between MTZ and NTR produces a toxic metabolite that causes targeted destruction of ventricular CMs (Zhang et al. 2013). As shown in Fig. 1, most of the ventricular CMs in the zebrafish embryos were destroyed 24 h after MTZ-mediated ablation (Fig. 1C) compared with those in the control group (Fig. 1B). The number of damaged ventricles increased at 48 (Fig. 1D) and 96 hpt (Fig. 1E), and failed to fully recover to the normal level. This indicates that the myocardial injury model in zebrafish was successfully constructed.

### EW promoted myocardial injury repair

EW has been shown to provide therapeutic benefits in cardiac diseases. However, its potential protective mechanism remains unclear. We investigated whether EW mediates its beneficial effects in CVDs by promoting CMs repair and regeneration. At 24 h after myocardial ablation, EW was administered for 3 days (Fig. 1A). The extent of myocardial regeneration and repair was measured at 48, 72, and 96 hpt (Fig. 1A) with MTZ. Twenty-four hours after MTZ treatment (24 hpt), the embryos were treated with 10, 25, and 50 μg/mL of EW to analyse cardiac regeneration (Fig. 1A). We found that the survival rate of zebrafish larvae in the 50 μg/mL group was very low (Fig. 1F). The ventricular regenerative ability in the 10 μg/mL treatment group was also markedly low (Fig. 1G). Therefore, after statistical analyses, 25 μg/mL EW was selected as the treatment dose for subsequent experiments. We found that the MTZ-induced ventricular ablation and pericardial oedema were markedly restored after treatment with 25 μg/mL EW. Morphological analyses showed that the zebrafish embryos in the MTZ group (Fig. S1A) exhibited pericardial oedema (marked by a black arrow) compared with those in the DMSO group (Fig. S1A), but these changes were reversed following EW treatment. The pericardial oedema rate in the MTZ model group was significantly higher than that in the DMSO group (Fig. S1B). EW can significantly reduce the pericardial edema rate caused by MTZ (Fig. S1B).

**Fig. 2:**
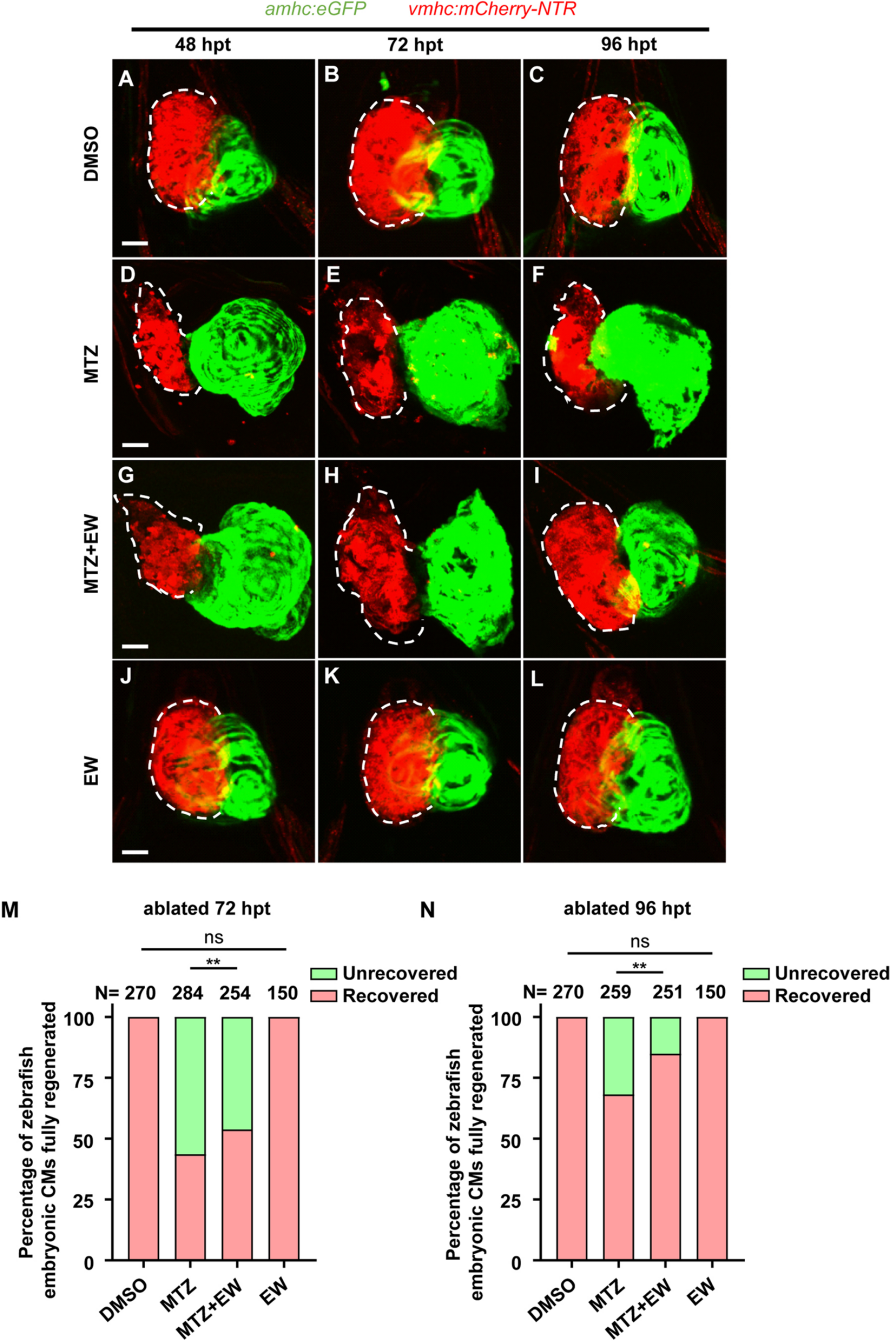
Eerdun-Wurile (EW) promotes myocardial injury repair. Representative images of ventricular morphology of embryos in the control (**A**-**C**), metronidazole (MTZ) ablation (**D**-**F**), EW treatment (**G**-**I**) groups and EW alone (**J**-**L**). Statistical analysis of myocardial regeneration at 72 hpt (**M**). Statistical analysis of myocardial regeneration at 96 hpt (**N**). **, *P* < 0.01. Scale bar, 20 μm

In control group, the ventricular morphology of zebrafish is intact (Fig. 2A-C). Compared with the regeneration of ventricles in the MTZ-induced ventricular ablation group (Fig. 2D-F), the regeneration in EW-treated embryos (Fig. 2G-I) increased significantly at 72 and 96 hpt. After EW treatment, the morphology of the repaired ventricular CMs (Fig. 2G-I) was similar to that of the DMSO control group (Fig. 2A-C). Meanwhile, EW alone did not have any effect on zebrafish embryos (Fig. 2J-L). Single slide images of the ventricular morphology besides the projections have been supplied in the supplementary materials (Movie S1– 12). Statistical analysis showed that the percentage of zebrafish with complete regeneration of damaged ventricles in the EW-treated group at 72 (Fig. 2M) and 96 hpt (Fig. 2N) was significantly higher than that in the MTZ model group. These results suggested that the Mongolian medicine EW promotes ventricular regeneration in zebrafish.

### EW reduced the death of myocardial cells

**Fig. 3:**
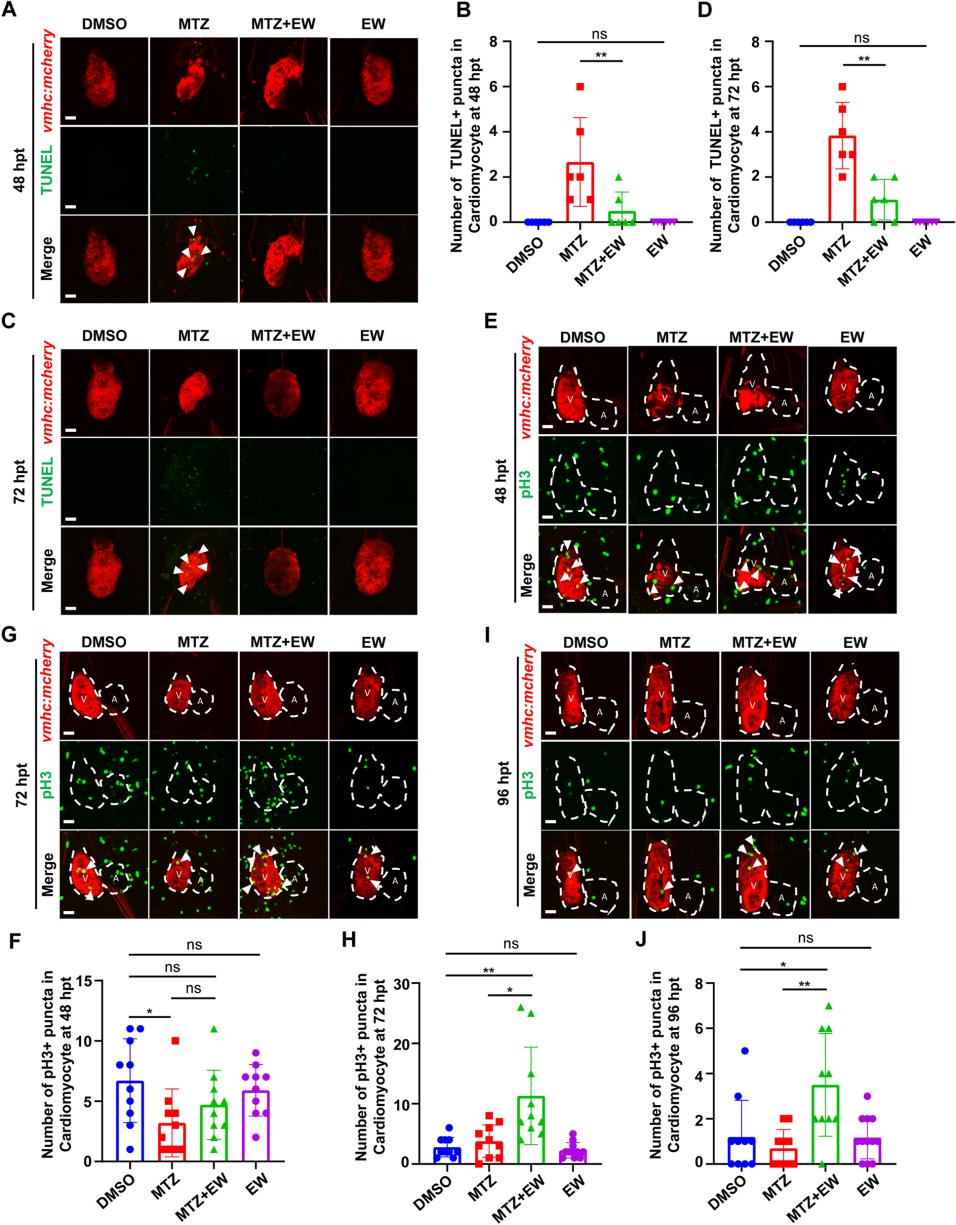
Eerdun-Wurile (EW) reduces apoptosis of cardiomyocytes and promotes proliferation of cardiomyocytes. Representative images of TUNEL staining in the control, metronidazole (MTZ) ablation, EW treatment groups and EW alone at 48 hpt (**A**), 72 hpt (**C**). White arrows indicate TUNEL positive (TUNEL +) cardiomyocytes (CMs). Statistical analysis of apoptosis cardiomyocyte at 48 (**B**), 72 hpt (**D**). Immunofluorescent analysis of cardiomyocytes proliferation at 48 (**E**), 72 (**G**), and 96 hpt (**I**). White arrows indicate phosphorylated histone H3 positive (pH3 +) cardiomyocytes (CMs). Statistical analysis of cardiomyocytes proliferation at 48 (**F**), 72 (**H**), and 96 hpt (**J**). *, *P* < 0.05; **, *P* < 0.01. Scale bar, 20 μm

Apoptosis of cardiomyocytes after myocardial injury is the main clinical manifestation of myocardial infarction. Therefore, we conducted TUNEL staining to evaluate cardiomyocytes apoptosis. At 48 hpt, a significant rise apoptotic signals in the MTZ model group could be detected (Fig. 3A, B), compared to that in DMSO. And in EW-treated embryos, the apoptotic signals decreased largely (Fig. 3A, B). And in 72 hpt, EW could reduce cardiomyocytes death when added with EW (Fig. 3C, D).

### EW promoted myocardial cell proliferation

Cell proliferation is an important step in CM regeneration. We found that treatment with EW promotes heart regeneration in zebrafish after ventricular injury. Therefore, we analysed whether EW enhances the rate of cell proliferation. We used pH3 to label cells at the G2-M mitotic stage and performed whole-mount immunofluorescence staining to observe cell proliferation. At 48 hpt (Fig. 3E, F), the proliferation rate of zebrafish embryonic cells was not significantly different between the MTZ and EW groups. However, at 72 (Fig. 3G, H) and 96 hpt (Fig. 3I, J), the number of CMs that proliferated in the EW treatment group was significantly higher than that in the MTZ ablation group. Results of the statistical analyses (Fig. 3F, H, J) showed that EW significantly enhanced cell proliferation in the injured area at 72 and 96 hpt compared with that in the control group. These data suggest that EW promotes the proliferation of myocardial cells and leads to heart regeneration in zebrafish.

### EW significantly reduced oxidative stress

We observed increased CM death and severe inflammatory reactions following heart injury in the zebrafish. Inflammation is closely related to oxidative stress. Studies have shown that ROS leads to abnormal translation and activation of ribosomal toxic reactions, which damage the cellular structure and integrity (Snieckute et al. 2023). Therefore, we analysed the expression of ROS in zebrafish following injury to the heart and EW treatment.

The production of ROS in zebrafish was measured at 72 and 96 hpt. The expression of ROS in the MTZ ablation group (Fig. S2B, G) was significantly higher than that in the normal group (Fig. S2A, F); however, after EW treatment (Fig. S2C, H), the expression of ROS significantly reduced. Thus, EW significantly reduced ROS production induced by myocardial cell injury.

### EW effectively restored heart function in zebrafish

**Fig. 4:**
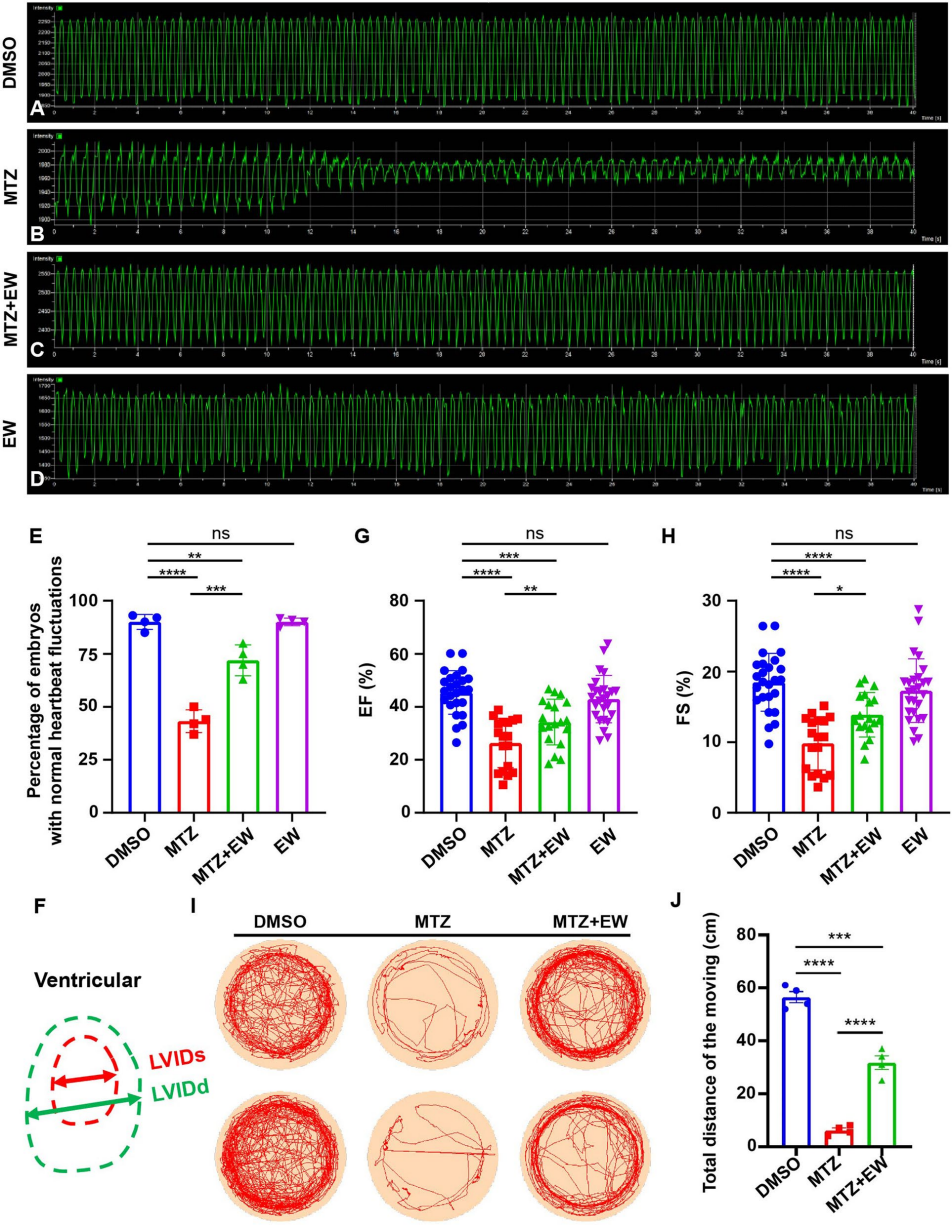
Eerdun-Wurile (EW) effectively restores heart function in zebrafish. Representative heartbeat fluctuations charts of zebrafish in control (**A**), metronidazole (MTZ) ventricular ablation (**B**), EW treatment (**C**) groups, and EW alone group (**D**). Statistical analysis of the proportion of embryos with normal heartbeat fluctuations (**E**). **F** Schematic presentation of the measuring method of end diastolic ventricular inner diameters (LVIDd) and end systolic ventricular inner diameters (LVIDs). Analyses of EF (**G**) and FS (**H**) in different groups. **I** Representative traces of individual zebrafish from different groups during the thigmotaxis test. **J** Statistical analysis of the movement trajectories *, *P* < 0.05; **, *P* < 0.01; ***, *P* < 0.001, ****, *P* < 0.0001.

Heartbeat fluctuation frequency and contractibility are the important index of cardiac function (Berntson et al. 1997). We assessed cardiac function using confocal microscopy to record the real-time heartbeat fluctuation of embryos. The heartbeat fluctuation of zebrafish larvae after EW treatment was detected at 72 hpt. We found that compared with that in the normal group (Fig. 4A), the heartbeat frequency in the MTZ ablation group (Fig. 4B) decreased, and the fluctuation amplitude was abnormal. However, the abnormal heartbeat fluctuation significantly improved following EW treatment (Fig. 4C). In the EW alone group, there was no difference in heartbeat fluctuations compared to the DMSO group, both in terms of the frequency and amplitude (Fig. 4D). We counted the proportion of embryos with normal frequency and heartbeat amplitude in each group. After EW treatment, the proportion of embryos with normal heartbeat fluctuations has basically returned to normal levels (Fig. 4E). Thus, EW effectively restored heart function and relieved arrhythmia. To evaluate the ability of the heart contractibility, end diastolic and end systolic ventricular inner diameters (LVIDd, LVIDs) were measured (Fig. 4F), refer to the evaluation methods of humans and mammals. The systolic function of MTZ ablation group hearts was significantly impaired versus control group, ejection fraction (EF) 26% versus 46% (Fig. 4G), fractional shortening (FS) 10% versus 18% (Fig. 4H). The EW treatment group exhibited a gradual increase of EF (34%) (Fig. 4G) and FS (14%) (Fig. 4H), suggesting the great progression in heart contractibility. Therefore, EW effectively restored heart function.

Heart function is also closely related to locomotor activity. Therefore, we tested the locomotor activity of zebrafish embryos to determine the therapeutic effect of EW on heart function. The locomotor activity of embryos with ventricular ablation was significantly reduced compared with that in the normal group, whereas the embryos with heart damage remained in their original position for an extended period (Fig. 4I, J). Following EW treatment, the locomotor activity of the embryos recovered significantly, and the distance moved significantly increased within 15 min, and was almost equal to that of the normal group (Fig. 4I, J). Statistical analysis of the data revealed (Fig. 4E, G, H, J) that EW improved heart function and motor delay caused by heart damage.

### Transcriptome sequencing

**Fig. 5:**
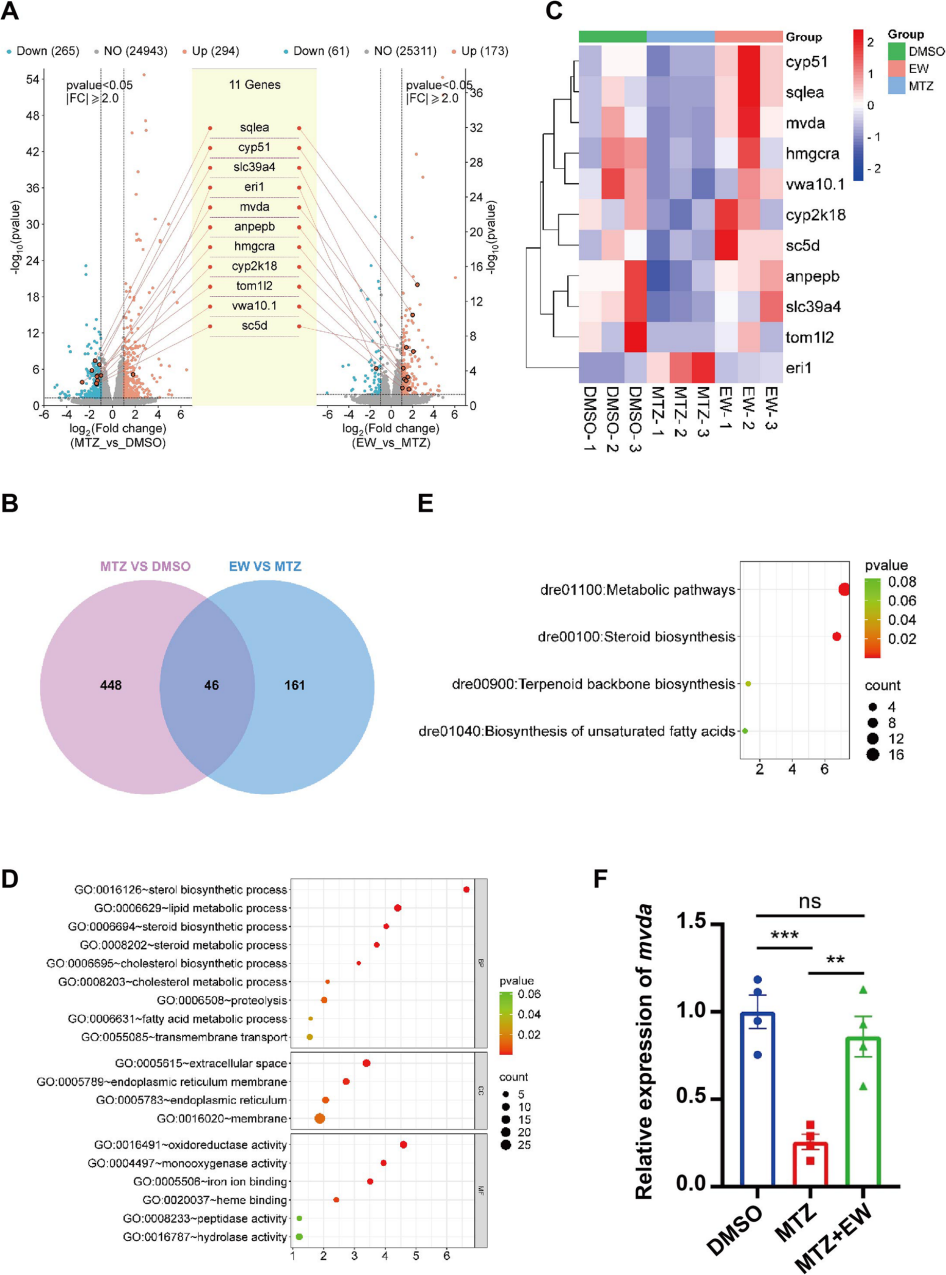
Transcriptome sequencing. **A** Heat map showing all the differentially expressed genes (DEGs) identified by RNA-sequencing. **B** Volcano plot showing the differentially expressed genes (DEGs) (criteria: *P* < 0.05, Log_2_FC fold change > 1 or < -1) altered in metronidazole (MTZ) group and the Eerdun-Wurile (EW) treatment group at 48 hpt. **C** Venn plot showing the strategy for screening the opposite DEGs in MTZ and EW group. Gene ontology (GO) (**D**) and Kyoto Encyclopedia of Genes and Genomes (KEGG) (**E**) analysis show the enrichment results of 46 opposite DEGs obtained in (**C**). **F** Quantitative PCR analysis was performed to confirm the changes in mevalonate diphosphate decarboxylase a (*mvda*) expression in the different groups (RNA-sequencing results). **, *P* < 0.01; ***, *P* < 0.001

To further examine the mechanism by which EW promoted myocardial injury repair, we conducted transcriptome sequencing. Transcriptome sequencing showed that the differentially expressed genes (DEGs) related to heart regeneration decreased after heart injury and recovered after EW treatment (Fig. 5A). Compared with DMSO control group, the MTZ injury group had 294 upregulated and 265 downregulated DEGs (criteria: *P* < 0.05, Log_2_FC fold change > 1 or < -1, Fig. 5A). Meanwhile compared with MTZ injury group, the EW treatment group had 173 upregulated and 61 downregulated DEGs (criteria: *P* < 0.05, Log_2_FC fold change > 1 or < -1, Fig. 5A). Forty-six differentially expressed genes (DEGs), including *cyp51, sqlea, mvda, hmgcra, vwa10.1, cyp2k18, sc5d, anpepb, slc39a4, tom1l2, *and* eri1* were identified based on the screening criteria (Fig. 5B). These DEGs have different changing trends in different groups, either upregulate first and then downregulate, or downregulate first and then upregulate (Fig. 5C). We used the 46 DEGs to conduct bioinformatics analyses, including Gene Ontology (GO) analysis (Fig. 5D), Kyoto Encyclopedia of Genes and Genomes (KEGG) analysis (Fig. 5E). Interestingly, the two types of bioinformatics analyses showed some same pathways enriched, mainly involved steroid biosynthesis, metabolic, unsaturated fatty acid biosynthesis, and terpenoid skeleton biosynthesis pathways. Metabolic and lipid metabolism pathways accounted for a large proportion of associated pathways. Therefore, we hypothesised that the regeneration of the zebrafish myocardium is regulated by metabolic pathways.

Among the DEGs, the changes in *mvda* expression caught our attention. *mvda* expression decreased after ventricular ablation and increased after EW treatment (Fig. 5A, C). Therefore, we hypothesised that changes in *mvda* expression regulate the rate of heart regeneration in zebrafish. To verify the changes in *mvda* gene expression identified by the transcriptome analyses, we used real-time PCR to analyse *mvda* expression in the different groups. The real-time PCR results showed that the *mvda* expression levels decreased after MTZ treatment, and restored after EW treatment at 48 hpt (Fig. 5F).

To reveal the *mvda* function in whole course of heart regeneration, we examined *mvda* expression at sequential regeneration time points at 24, 48, 72, and 96 hpt (Fig. S3). According to the real-time PCR results, we found that *mvda* expression was significantly downregulated at all time points after ventricular injury in the MTZ group (Fig. S3A-D). EW significantly upregulated *mvda* in the early stage of regeneration (48 hpt, Fig. S3B). But in the late stage of regeneration (72 hpt and 96 hpt, Fig. S3C, D), the expression of *mvda* returned to its damaged state level. Whole mount in situ hybridization had been performed to examine *mvda* expression at 48 hpt. Mvda was expressed ubiquitously, with relatively high expression in the liver region (Fig. S3E). To further confirm whether *mvda* is specifically expressed in the cardiomyocytes, the double immunofluorescence (mCherry for ventricle cardiomyocyte and Alex488 for MVDA antibody) had been performed to confirm mvda co-localization with cardiomyocyte markers. The MVDA is expressed specifically in the atria (Fig. S4).

### *mvda* knockdown affected CM regeneration in zebrafish

Transcriptome sequencing revealed that the expression of *mvda* decreased after heart injury and increased after EW treatment. Therefore, we aimed to investigate whether the knockdown *mvda* genes attenuated heart regeneration, and conversely, overexpression of *mvda* promoted heart regeneration. We performed microinjection of *mvda* ATG morpholino (MO), which would suppress translation of *mvda* mRNA before ventricular ablation. To validate the efficacy of *mvda* MO, a 60 bp fragment including *mvda* ATG MO binding site of zebrafish *mvda* gene was fused to the N-terminal of EGFP (Fig. S5E). After co-injection with *mvda* ATG MO, the expression of EGFP was successfully blocked (Fig. S5A-D). The *mvda* expression level indeed decreased when injected *mvda* MO (Fig. S5F).

**Fig. 6:**
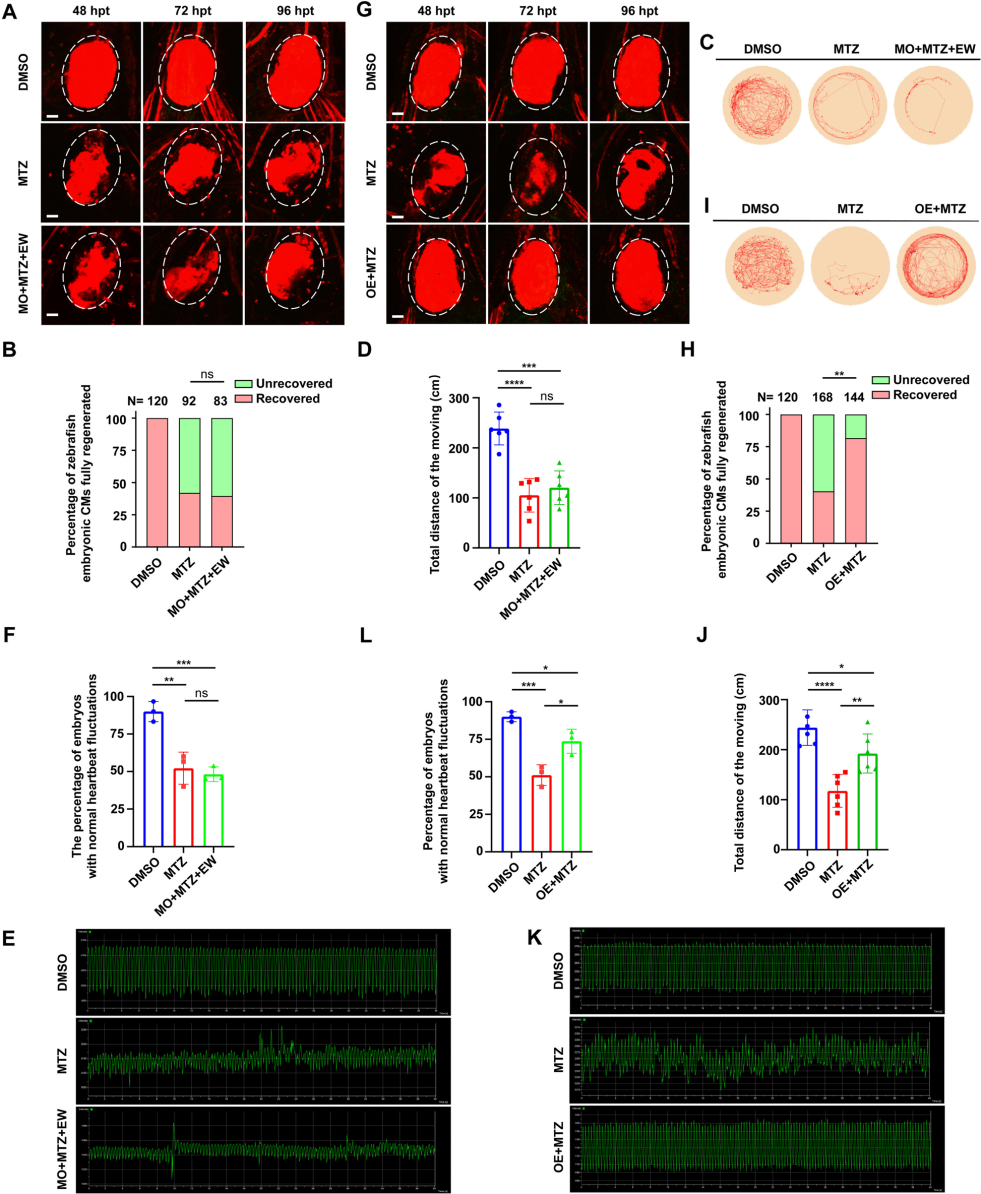
*mvda* knockdown/ overexpression affects heart regeneration in zebrafish. Representative images of ventricular morphology of zebrafish in DMSO, MTZ, and MO + MTZ + EW (**A**). Statistical analysis of ventricular regeneration (**B**). **C** Representative traces of individual zebrafish from different groups during the thigmotaxis test. **D** Statistical analysis of the movement trajectories. Representative heartbeat fluctuations charts of zebrafish in DMSO, MTZ, MO + MTZ + EW (**E**). Statistical analysis of the proportion of embryos with normal heartbeat fluctuations (**F**). Representative images of ventricular morphology of zebrafish in DMSO, MTZ, OE + MTZ (**G**). Statistical analysis of ventricular regeneration (**H**). **I** Representative traces of individual zebrafish from different groups during the thigmotaxis test. **J** Statistical analysis of the movement trajectories. Representative heartbeat fluctuations charts of zebrafish in DMSO, MTZ, OE + MTZ (**K**). Statistical analysis of the proportion of embryos with normal heartbeat fluctuations (**L**). *, *P* < 0.05; **, *P* < 0.01; ***, *P* < 0.001, ****, *P* < 0.0001. Scale bar: 20 μm

**Fig. 7:**
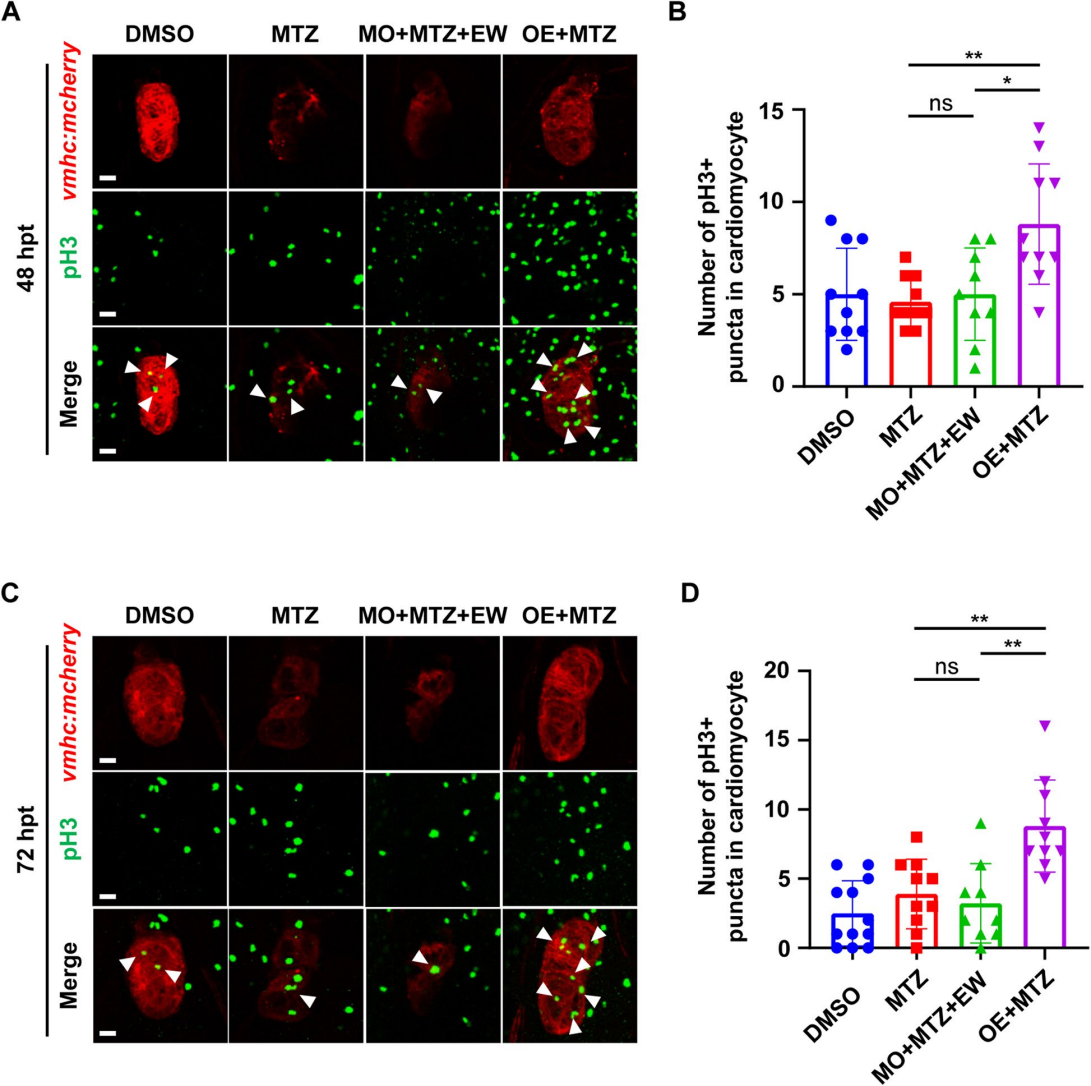
*mvda* affects the proliferation of cardiomyocytes. Immunofluorescent analysis of cardiomyocytes proliferation at 48 (**A**), 72 hpt (**C**) in *mvda* overexpression and knocking down group. White arrows indicate phosphorylated histone H3 positive (pH3 +) cardiomyocytes (CMs). Statistical analysis of cardiomyocytes proliferation at 48 (**B**), 72 hpt (**D**),. *, *P* < 0.05; **, *P* < 0.01. Scale bar, 20 μm

We found that following morpholino-mediated knockdown of *mvda*, EW treatment did not promote heart regeneration efficiently (Fig. 6). The ventricular regeneration rate of the EW treatment (Fig. 6A) decreased significantly following *mvda* knockdown (Fig. 6B). Single slide images of the ventricular morphology besides the projections have been supplied in the supplementary materials (Movie S13-17), (Movie S18-24). Thus, morphological analyses of the zebrafish revealed that EW failed to relieve ventricular ablation-induced pericardial oedema following *mvda* knockdown (Figure S6A-S6B). The total moving distance and locomotor activity were significantly reduced, blocking the therapeutic effect of EW (Fig. 6C, D). The total moving distance and locomotor activity were even lower than those in the MTZ model group, when knocking down *mvda* by morpholino (MO) (Fig. 6C, D). The heartbeat fluctuation frequency also cannot be restored either, when using MO to knock down *mvda* (Fig. 6E, F). The proliferation of cardiomyocytes seems to pause after MVDA knockdown at 48 (Fig. 7A, B) and 72 hpt (Fig. 7C, D) compared with that in EW treatment group.

### *mvda* overexpression promoted CM regeneration

Given that *mvda* knockdown inhibited heart regeneration after ventricular ablation in zebrafish, we tested the hypothesis that overexpression of *mvda* (OE) promotes heart regeneration after ventricular ablation in the absence of EW treatment. We conducted *mvda* expression plasmid (Fig. S7A-C). *mvda* mRNA was microinjected into fertilised zebrafish embryos at the one-cell stage, and the expression level of *mvda* significantly increased by real time PCR verification (Fig. S7D).

The results showed rapid regeneration of the zebrafish heart following *mvda* overexpression, even in the absence of EW (Fig. 6G), with restoration of the normal architecture of the tissue (Fig. S6D-F). However, the embryos without *mvda* overexpression exhibited slow ventricular regeneration after ablation (Fig. 6G). Single slide images of the ventricular morphology besides the projections have been supplied in the supplementary materials (Movie S18–24), (Movie S25-30). The overexpression of *mvda* was beneficial for CM regeneration in zebrafish (Fig. 6H). Morphological analyses of the zebrafish revealed that the MTZ-induced pericardial oedema was rapidly relieved in zebrafish overexpressing *mvda*, even in the absence of EW treatment (white arrows) (Fig. S6D-F). In contrast, ventricular ablation-induced pericardial oedema persisted in zebrafish without *mvda* overexpression (Fig. S6D-F). The locomotor activity and the heartbeat fluctuation frequency were restored markedly, when *mvda* overexpression (Fig. 6I-L). The proliferation of cardiomyocytes was significantly enhanced after overexpression of *mvda* (Fig. 7).

To strength the study, we used the cardiomyocyte-specific genetic tools for overexpressing *mvda *(Fig. S8A). When overexpression *mvda* by Tol2 system in cardiomyocytes, heart regeneration could also be recovered efficiently after ventricular ablation (Fig. S8B-H).

## Discussion

In this study, we found that the traditional Mongolian medicine EW promoted myocardial regeneration in zebrafish. EW induced CMs to re-enter the cell cycle, proliferate, and regenerate to repair damaged ventricles. These regenerated myocardial cells restore cardiac function. Knockdown and overexpression studies confirmed that EW promoted myocardial regeneration by regulating the *mvda* gene.

Zebrafish are important model organisms for the study of human diseases (Varshney & Burgess 2014). In the present study, we used a zebrafish ventricular ablation system and found that EW effectively promoted myocardial cell proliferation and ventricular regeneration after ventricular ablation, and improved bradycardia caused by arrhythmia and heart damage in zebrafish. Compared with other mammalian models, zebrafish has strong regenerative abilities and transparent embryos allowing for real-time monitoring of the heart development (Yalcin et al. 2017). Therefore, zebrafish has become a powerful and versatile model to study CVDs (Gore et al. 2018).

Cardiac regeneration involves intrinsically responsive substrates and external stimuli. Understanding the molecular mechanisms and differences in cardiac regeneration between vertebrates and mammals is very important for developing new therapies for CM regeneration. BMP7 overexpression promotes CM regeneration in zebrafish and adult mice (Bongiovanni et al. 2024). Versican (a cardiac fibroblast–derived extracellular matrix component) also promotes CM proliferation and cardiac repair. In adult mice, the intramyocardial injection of versican following MI enhanced CM proliferation, reduced fibrosis, and improved cardiac function (Feng et al. 2024). Leucine-rich repeat-containing 10 (Lrrc10), a component of the cardiac dyad, functions as a negative regulator of proliferation, prevents cardiomegaly, and induces redifferentiation (Nguyen et al. 2023).

We found that EW stimulated heart regeneration by regulating *mvda*. *mvda* expression decreased following heart injury. EW treatment markedly increased *mvda* expression and ventricular regeneration. MVDA enables diphosphomevalonate decarboxylase activity and is involved in isopentenyl diphosphate biosynthesis in the mevalonate pathway. The MVDA pathway may be upstream of or within the cholesterol metabolic pathway. The human homolog of zebrafish MVDA is mevalonate diphosphate decarboxylase (MVD). Previous studies have found that MVDA is necessary for the early development of zebrafish and affects the development of the heart, eyes, and tail; however, the specific mechanism remains largely unknown (Wong et al. 2021). Our study confirmed the positive role of MVDA in heart regeneration.

Studies have shown that human newborns have strong myocardial regenerative ability following MI (Haubner et al. 2016). Notably, the maturation of newborn myocardial cells is characterised by a metabolic transformation from glycolysis to fatty acid oxidation, which is an impediment to the regeneration of adult myocardial cells (Maroli & Braun 2021; Yuan & Braun 2017). By eliminating fatty acid oxidation, myocardial cells can be transformed into an immature state, which promotes myocardial cell proliferation and heart regeneration (Li et al. 2023). Deletion of pyruvate dehydrogenase-4 and inhibition of succinate dehydrogenase to promote anaerobic metabolism promote the proliferation of myocardial cells (Bae et al. 2021; Cardoso et al. 2020; Magadum et al. 2020). In addition, decreased expression of lipoprotein lipase (LpL) on the surface of myocardial cells affects the uptake of fatty acids by myocardial cells, whereas overexpression of LpL increases the uptake of fatty acids and leads to cardiomyopathy (Yagyu et al. 2003).

Previous studies have shown that sphingolipid metabolism controls regeneration of the mammalian heart. Sphingosine-1-phosphate (S1P) enzymes (SphK1 and SphK2) regulate cardiac regeneration. Reactivation of SphK2 induces the cell cycle re-entry of adult CMs and enhances regeneration (Ji et al. 2024).

These studies suggest that metabolic transformation can transform myocardial cells into an immature state, thereby promoting their proliferation and facilitating heart regeneration. In the present study, EW treatment was accompanied by metabolic transformation. We hypothesise that MVDA promotes the proliferation of myocardial cells through metabolic transformation; however, the specific mechanism requires further investigation. The bioactive chemicals in EW also need to be identified. This is an area to be restored in the later stages of our study.

## Materials and methods

### Zebrafish husbandry and maintenance

The zebrafish wild-type TU/WT, Tg (*amhc:eGFP)* and Tg (*vmhc:mCherry-NTR*) transgenic fish lines were raised in 10 L glass jars at a density of approximately 20 fish/jar. The Tg (*amhc:eGFP) and* Tg (*vmhc:mCherry-NTR*) transgenic fish line was a kind gift from Professor Ruilin Zhang at the Wuhan University (Zhang et al. 2013). The zebrafish were maintained in a circulating water filtration system at a pH of 7.0 ± 1.0, water temperature of 28 ± 1 ℃, and a 12-h light/dark cycle. The zebrafish were fed shrimp twice daily. The night before the experiment, the male and female fish were placed in the same incubator and separated by a comb. At 8:30 am on the following day, the zebrafish began to spawn. Embryos were collected in 0.005% 1-phenyl-2-thiourea (PTU) E3 medium (egg water). PTU solution inhibits the production of melanin and renders the bodies of the zebrafish larvae transparent, allowing for visual analysis of embryonic development. The zebrafish used in this study were maintained in the model animal platform at the Centre of Translational Medicine in Baotou Medical College. This study was reviewed and approved by the Experimental Animal Ethics Committee of the Baotou Medical College, Inner Mongolia University of Science and Technology, Inner Mongolia, China.

### Establishment of zebrafish myocardial injury regeneration model

The zebrafish transgenic line Tg (*vmhc:mCherry-NTR*) was mated with wild-type TU/WT. Three days after fertilisation, zebrafish embryos were treated with 5 mmol/L metronidazole (MTZ, Cat. No.: A600633; Sangon Biotech, Shanghai, China) for 4 h and then washed four times with the embryo culture solution. Myocardial injury was observed at 24, 48, and 96 h post-treatment (hpt) with MTZ using a laser confocal microscope. The regeneration ratio was calculated as the number of recovered larvae over the number of total injured larvae (Wang et al. 2021; Yu et al. 2024).

### Drug preparation

Eerdun-Wurile (EW) was purchased from the Affiliated Hospital of Inner Mongolia Minzu University, China. Dissolved 0.2 g of EW drug in 10 ml of 30% DMSO and sonicated. The storage concentration is 20 mg/ml. The usage concentration is 25 μg/mL, and ultrasound is required before each use.

### Real-time observation of myocardial injury repair in zebrafish embryo

Myocardial regeneration was observed after 24 hpt with MTZ and 25 μg/mL EW treatment (Fig. 1A). The zebrafish embryos were anaesthetised with tricaine (0.08% by volume), mounted in a low-melting agarose gel (0.5%), and live imaging of myocardial regeneration was performed using a confocal microscope (A1 + confocal microscope, Nikon, Japan).

### Immunofluorescent staining of phosphorylated histone H3 (pH3) and MVD

Embryos were fixed with 4% paraformaldehyde overnight, washed with PBST (PBS containing 0.1% Tween 20), dehydrated with methanol, and digested with protease K (100 mg/L). Samples were blocked with blocking buffer (10% goat serum in PBS containing 0.5% Triton X-100) for 2 h at 22~25℃ and then incubated with a primary antibody against pH3 (1:500; Cat. No.: 3377; Cell Signalling Technology, Boston, USA) and MVD (1:500, Cat.No.:PA5-22,164; Invitrogen) overnight. The samples were incubated with an Alexa Fluor 488-conjugated secondary antibody (1:500; Cat. No.: A11029; Invitrogen, California, USA). The reaction was terminated with PBST. Images were captured using an A1 + confocal microscope (Nikon). The experiment was repeated at least thrice.

### Measurement of reactive oxygen species (ROS)

Zebrafish embryos belonging to the four groups were placed in six-well plates (10 embryos/well). DCFH-DA fluorescent probe (1:1000, Cat. No.: S31479, Shanghai Yuanye Biotechnology, Shanghai, China) was added to the wells, and the embryos were incubated in a dark incubator for 1~2 h. Images were captured with a fluorescent microscope, and statistical analysis was performed.

### Measurement of zebrafish heart rate and heart contractibility

Zebrafish embryos were mounted in 0.5% low-melting agarose and imaged with a Nikon A1 + confocal microscope (20X water-immersion objective) in the ventral position. Zebrafish were imaged continuously for 40 s using a confocal microscope, and the heartbeat fluctuation frequency was measured and statistically analysed. End diastolic and end systolic ventricular inner diameters (LVIDd, LVIDs) were measured and fractional shortening (FS) and ejection fraction (EF) were calculated using the following equations (She et al. 2025): FS = (LVIDd − LVIDs) / LVIDd × 100%, EF = [(LVIDd)^3^ − (LVIDs)^3^] (LVIDd)^3^ × 100%.

### Analysis of zebrafish behaviour

Embryos were placed in a 24-well plate containing zebrafish culture solution (1 mL/well). Zebrafish embryo movement was recorded for 15 min using a behavioural instrument (Noldus, Holland). EthoVision® XT software was used to determine the motion-speed parameters. The experiment was repeated at least thrice.

### Transcriptome sequencing

Transcriptome sequencing of zebrafish embryos in the three groups was performed by Shanghai Applied Protein Technology (Shanghai, China), and changes in the differentially expressed genes (DEGs) were analysed. The sequencing data had been uploaded to Gene Expression Omnibus (GEO) database (accession number GSE287547).

### Real-time quantitative polymerase chain reaction (PCR) analysis

Total mRNA was extracted at different time points from embryos belonging to the dimethyl sulfoxide (DMSO), MTZ, MTZ + EW and EW groups. cDNA was synthesised using the PrimeScript® Reagent Kit (Cat. No.: K1622; Thermo, USA). *mvda* expression was detected by real-time quantitative PCR using the SYBR® Green Mix (Cat No.: 11202ES08; Shanghai Yisheng Bio., Shanghai, China). The data were normalised to β-actin expression. The gene primers used are listed in Fig. S9. The experiment was repeated at least thrice.

### Knockdown of *mvda* using morpholino oligonucleotides (MOs)

The gene *mvda* was silenced by MO injection. The *mvda*-MO (5'-ATATTTTCCCATTTCGTATTTA-3') and control MOs (5'-GGTCAGCATTCAAGAGACCATGCAT-3') were purchased from GeneTools (Philomath, OR, USA). *mvda* MO (8 ng) was injected into the zebrafish embryo at the single-cell stage using a microinjector, and follow-up experiments were carried out.

### Plasmid construction

The zebrafish *mvda* gene fragment was inserted into the pCS2 + plasmid, and then pCS2 + containing *mvda* (*mvda*-pCS2 +) was transformed into *E. coli* and amplified. For construction of the *mvda* (1–60)-GFP reporter plasmid, the zebrafish cDNA (1–60 bp) of *mvda* gene was amplified and cloned into pCS2^+^-EGFP vector. The Tol2 backbone plasmid contained cardiomyocyte-specific promoter *cmlc2* was a kind gift from Professor Ruilin Zhang at the Wuhan University. To construct Tol2 transgenesis vectors (Tol2-*cmlc2* promoter-*mvda*), the zebrafish *mvda* was amplified from *mvda*-pCS2^+^ plasmid and inserted Tol2 backbone plasmid by the *cmlc2* promoter with *KpnI* and *ClaI* restriction site. Transient *mvda* transgene construct within Tol2 vectors (40 pg) was microinjected into one-cell-stage embryos with Tol2 transposase mRNA (50 pg).

### mvda overexpression

The plasmid *mvda*-pCS2 + was extracted and the mRNA sequence of *mvda* was synthesised using the mMACHINE® SP6 Transcription Kit (Cat No.: AM1340; Ambion, USA). *mvda* mRNA was microinjected into fertilised zebrafish embryos at the one-cell stage. Myocardial regeneration was observed at 3 days post fertilisation (dpf) after 4 h of MTZ treatment.

### TUNEL assay

Terminal transferase UTP nick end labeling (TUNEL) was performed with In Situ Cell Death Detection Kit and TMR Red Kit (Roche, 12,156,792,910) following manufacturer's instruction. Specifically, zebrafish at the indicated stages were fixed with 4% PFA, permeabilized with PBS/0.5% TritonX-100 and then incubated in TUNEL staining solution at 37 °C for 2 h.

### Statistical analyses

All data were statistically analysed using the GraphPad Prism 8 software with one way ANOVA. Statistical significance was set at *P* < 0.05. Error values were presented as the standard error of the mean (SEM). Each experiment was repeated at least thrice.

## Supplementary Information

Supplementary Material 1: Fig. S1. Morphological changes after cardiomyocyte injury and regeneration; Fig. S2. Eerdun-Wurile (EW) significantly reduces oxidative stress; Fig. S3. The expression of *mvda* in the whole course of heart regeneration; Fig. S4. Immunofluorescence expression of MVDA; Fig. S5. Validation of the efficacy of *mvda* morpholino oligonucleotides (MOs); Fig. S6. Morphological changes in *mvda* knocking down and overexpression groups; Fig. S7. Construction of *mvda* overexpression plasmid; Fig. S8. Cardiomyocyte-specific genetic tools for overexpressing *mvda*; Fig. S9. Primer Sequence.

Supplementary Material 2: Movie S1-3 showed all single slide images for the fluorescent image, related to figure 2A-2C. The ventricular morphology was showed at 48 (Movie S1), 72 (Movie S2) and 96 hpt (Movie S3) of DMSO group. Movie S4-6 showed all single slide images for the fluorescent image, related to figure 2D-2F. The ventricular morphology was showed at 48 (Movie S4), 72 (Movie S5) and 96 hpt (Movie S6) of MTZ group.

Supplementary Material 3: Movie S7-9 showed all single slide images for the fluorescent image, related to figure 2G-2I. The ventricular morphology was showed at 48 (Movie S7), 72 (Movie S8) and 96 hpt (Movie S9) of MTZ+EW group. Movie S10-12 showed all single slide images for the fluorescent image, related to figure 2J-2L. The ventricular morphology was showed at 48 (Movie S10), 72 (Movie S11) and 96 hpt (Movie S12) of EW alone group.

Supplementary Material 4: Movie S13-15 showed all single slide images for the fluorescent image, related to figure 6A. The ventricular morphology was showed at 48 (Movie S13), 72 (Movie S14) and 96 hpt (Movie S15) of DMSO group in figure 6A. Movie S16-18 showed all single slide images for the fluorescent image, related to figure 6A. The ventricular morphology was showed at 48 (Movie S16), 72 (Movie S17) and 96 hpt (Movie S18) of MTZ group in figure 6A.

Supplementary Material 5: Movie S19-21 showed all single slide images for the fluorescent image, related to figure 6A. The ventricular morphology was showed at 48 (Movie S19), 72 (Movie S20) and 96 hpt (Movie S21) of MO+MTZ+EW group in figure 6A. Movie S22-24 showed all single slide images for the fluorescent image, related to figure 6G. The ventricular morphology was showed at 48 (Movie S22), 72 (Movie S23) and 96 hpt (Movie S24) of DMSO group in figure 6G.

Supplementary Material 6: Movie S25-27 showed all single slide images for the fluorescent image, related to figure 6G. The ventricular morphology was showed at 48 (Movie S25), 72 (Movie S26) and 96 hpt (Movie S27) of MTZ group in figure 6G. Movie S28-30 showed all single slide images for the fluorescent image, related to figure 6G. The ventricular morphology was showed at 48 (Movie S28), 72 (Movie S29) and 96 hpt (Movie S30) of OE+MTZ group in figure 6G.

## Data Availability

The data used to support the findings of this study are included within the article.

## Abbreviations

MI: Myocardial infarction
EW: Eerdun Wurile
MVDA: mevalonate diphosphate decarboxylase a
CVD: Cardiovascular disease
CM: Cardiac Myocyte
MTZ: Metronidazole
NTR: Nitro reductase
EF: Ejection fraction
FS: Fractional shortening
DEGs: Differentially expressed genes
pH3: Phospho-Histone 3
KEGG: Kyoto Encyclopedia of Genes and Genomes
MO: morpholino
OE: Over expression
MVD: Mevalonate diphosphate decarboxylase
PTU: Phenyl-2-thiourea
Dpf: Days post fertilization
Hpt: Hours post treatment
ROS: Reactive oxygen species
DMSO: Dimethyl sulfoxide
