# Supplementary material for: Mongolian medicine Eerdun-Wurile promotes myocardial regeneration by regulating MVDA in zebrafish
Source: Cell Regen. 2025 Jun 6;14:22. doi: 10.1186/s13619-025-00235-z (PMC12143999; doi:10.1186/s13619-025-00235-z)
Supplement: Supplementary file 1 — Supplementary Material 1: Fig. S1. Morphological changes after cardiomyocyte injury and regeneration; Fig. S2. Eerdun-Wurile (EW) significantly reduces oxidative stress; Fig. S3. The expression of mvda in the whole course of heart regeneration; Fig. S4. Immunofluorescence expression of MVDA; Fig. S5. Validation of the efficacy of mvda morpholino oligonucleotides (MOs); Fig. S6. Morphological changes in mvda knocking down and overexpression groups; Fig. S7. Construction of mvda overexpression plasmid; Fig. S8. Cardiomyocyte-specific genetic tools for overexpressing mvda; Fig. S9. Primer Sequence. [file 13619_2025_235_MOESM1_ESM.docx]

Supplemental Materials

**Mongolian medicine Eerdun-Wurile promotes myocardial regeneration by regulating MVDA in zebrafish**

Xianghui Chen^1,2#^, Xiaoting Li^1,2#^, Jiajun Sun^1,2#^, Yufeng Lin^1,2,3^^#^, Yuanhao Li^1,2^, Xuehao Lv^1,2^, Rui Zhao^4^, Xinyue Gu^1^, Wenxuan Wang^1^, Yabin Xie^2,5^, Wei Xie^2,5^, Rengui Bade^2,5^, Shuyuan Jiang^2,5,6^, Xiaolei Liu^2,5,6^, Bo Zou^1,2^, Yannan Bi^1,2^, Guo Shao^2,5,7^, Haihua Bai^8^, Wei Zhu^2,6^*, Xiaoe Jia^1,2,5^*

Supplementary figures: S1-S9

Supplementary movies: S1-S30

**Figure S1. Morphological changes after cardiomyocyte injury and regeneration.** Representative morphological images of zebrafish (A). The percentage of pericardial edema in each group had been quantification (B). The black arrow indicates pericardial edema. ****, *P* < 0.0001 as compared with DMSO control group. ##, *P* < 0.01 as compared with MTZ group.
**Figure S2. Eerdun-Wurile (EW) significantly reduces oxidative stress.**


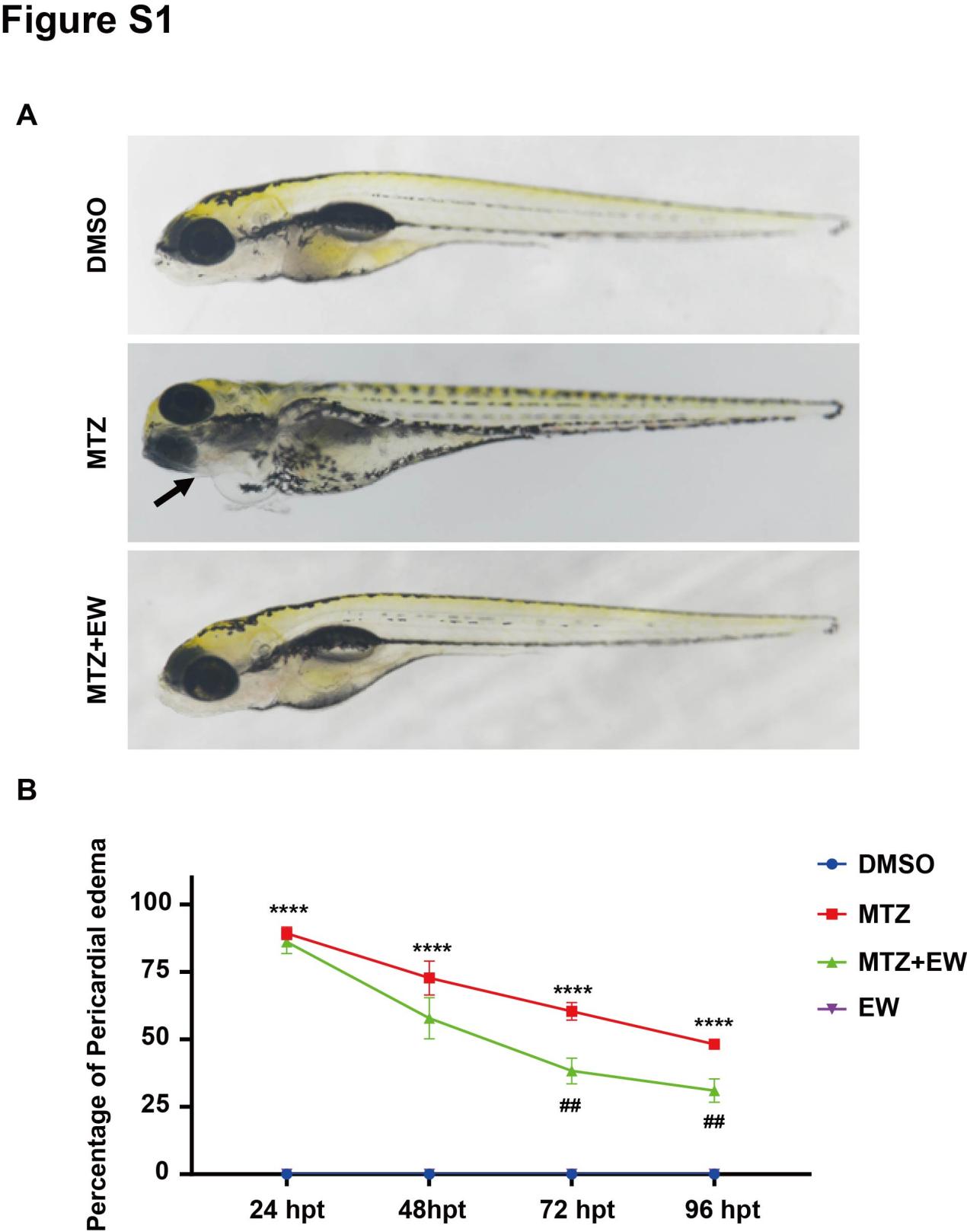

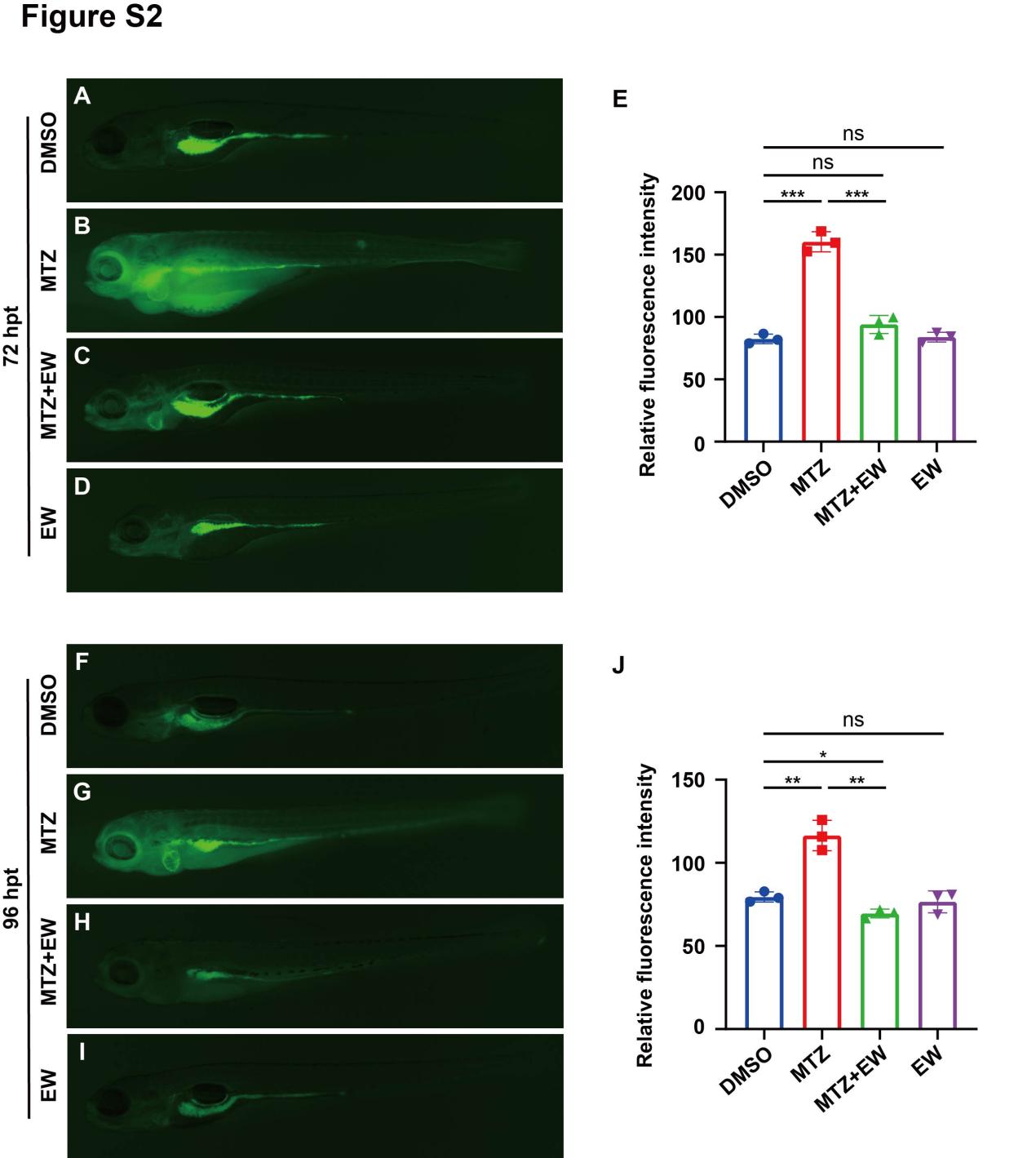


Representative reactive oxygen species (ROS) staining of embryos at 72 (A-D), 96 hpt (F-I). Statistical analysis of the fluorescence intensity at 72 (E), 96 hpt (J). *, *P* < 0.05; **, *P* < 0.01, ***, *P* < 0.001.


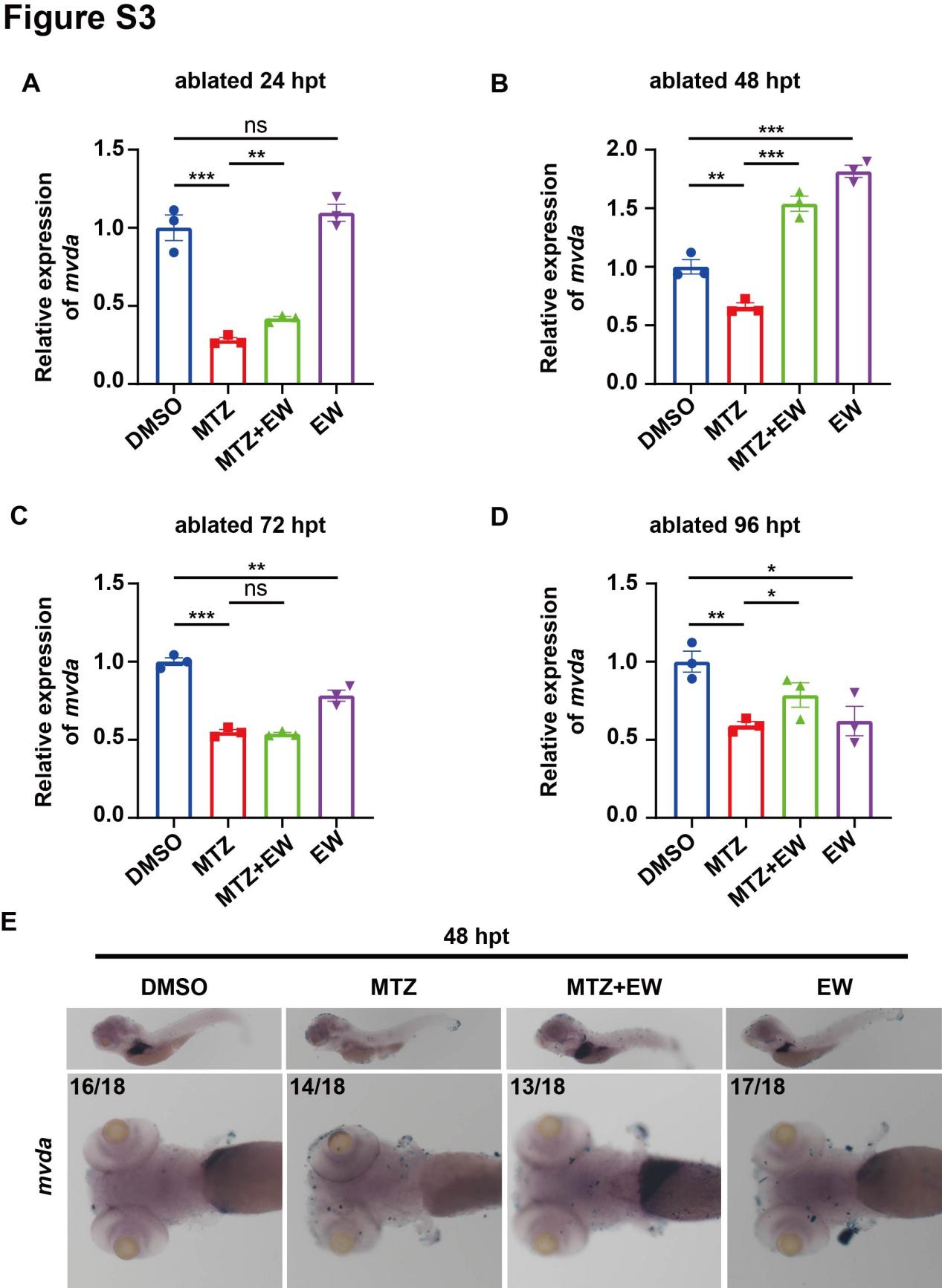


**Figure S3. The expression of *mvda* in the whole course of heart regeneration.**

(A-D) Quantitative PCR analysis was performed to confirm the changes in *mvda* expression in the different time course, at 24hpt (A), 48hpt (B), 72hpt (C), 96hpt (D). *, *P* < 0.05; **, *P* < 0.01; ***, *P* < 0.001. (E) Whole-mount *in situ* hybridization showing the expression level of *mvda* at 48hpt.


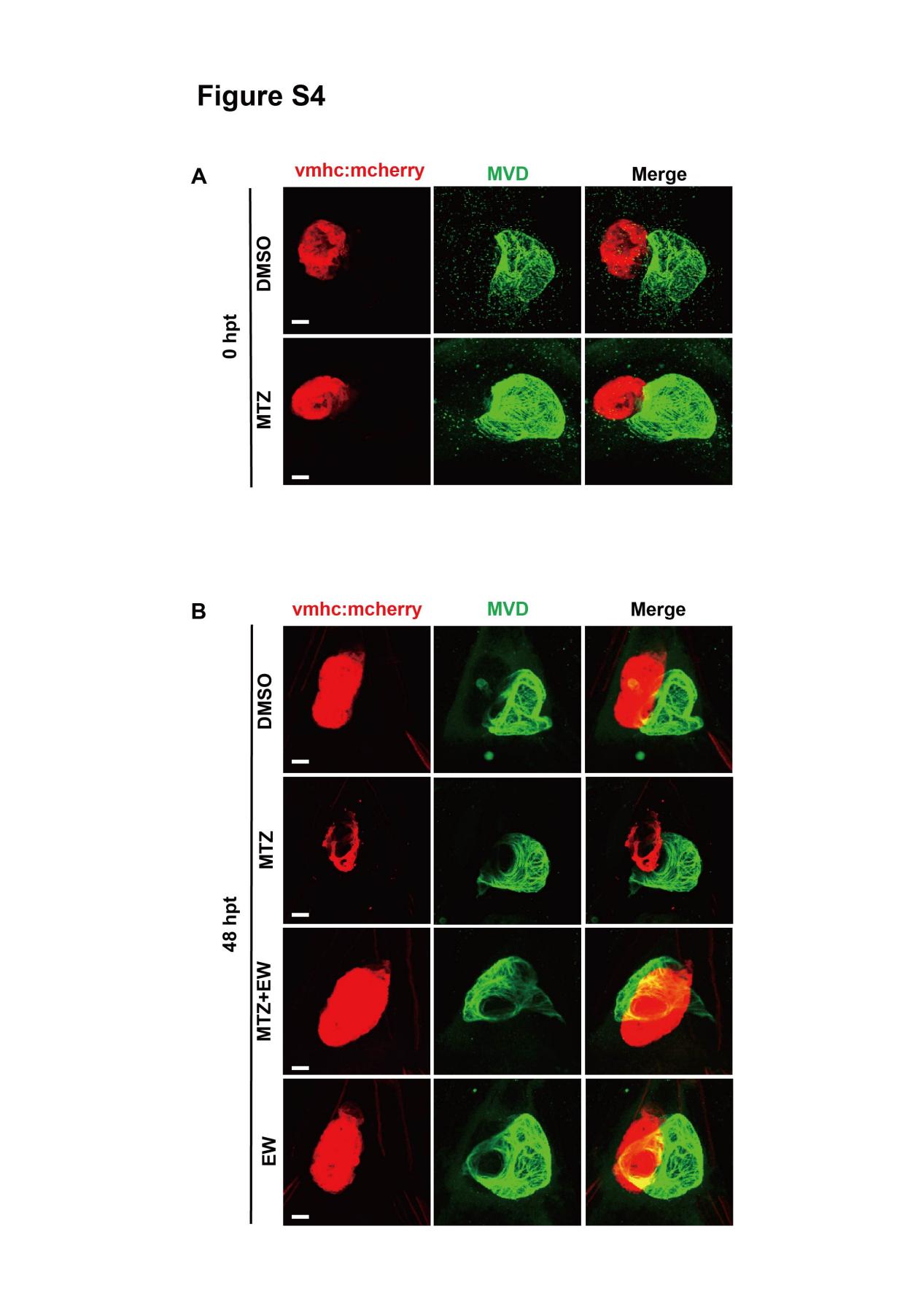


**Figure S4. Immunofluorescence expression of MVDA.**

Immunofluorescent analysis of MVDA at 0hpt in DMSO, MTZ (A). Immunofluorescent analysis of MVDA at 48hpt in DMSO, MTZ, MTZ+EW, and EW (B).


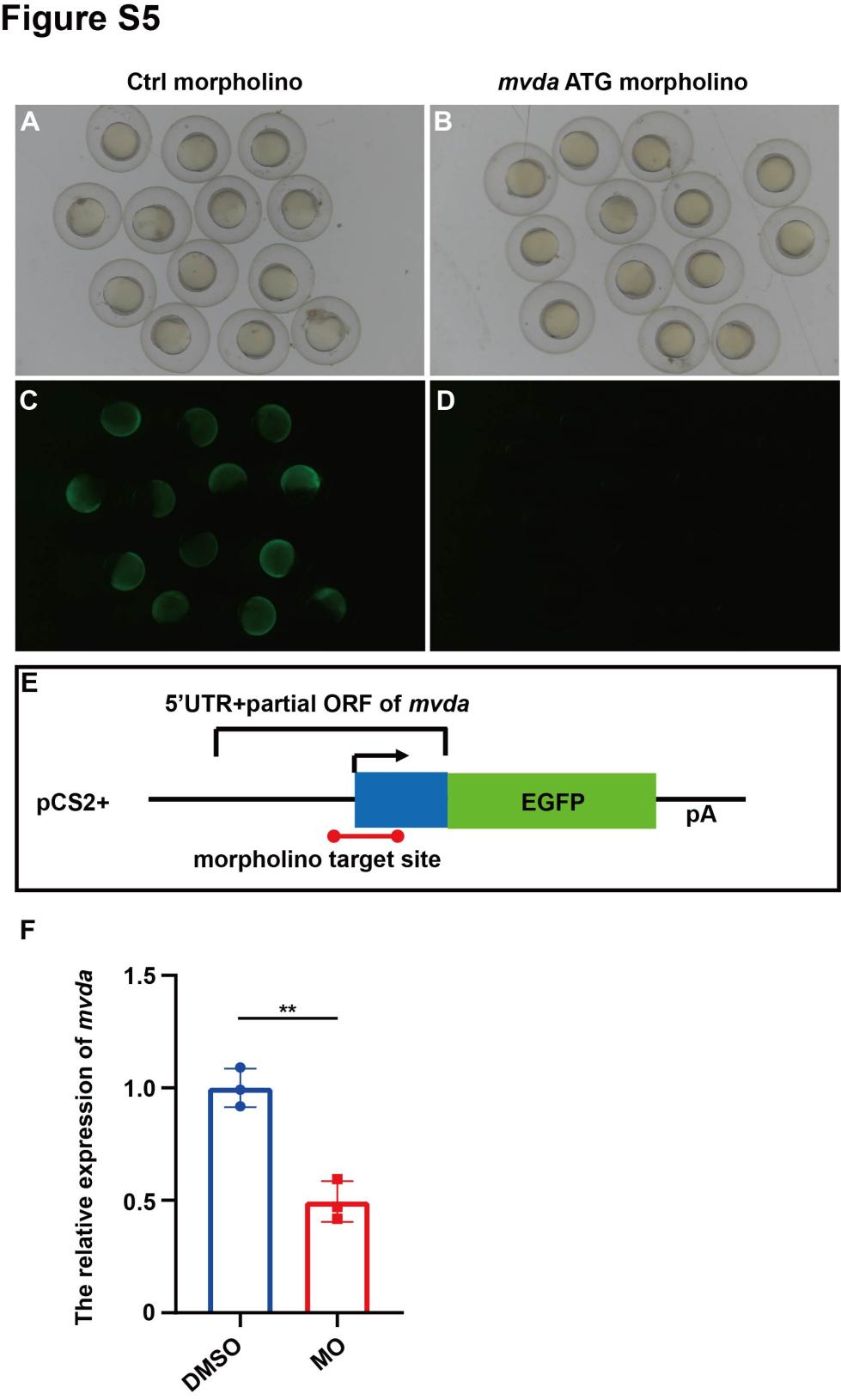


**Figure S5. Validation of the efficacy of *mvda* morpholino oligonucleotides (MOs).**

(A-D) Bright field and fluorescent images of zebrafish embryos at 20 hpf after injection of *mvda*-GFP reporter mRNA and control MO or *mvda* MO respectively at one-cell stage. (E) Schematic presentation of the *mvda*-GFP reporter plasmid used to test the knockdown efficiency of MO. The 5’ UTR and partial open reading frame (ORF) of *mvda* gene (blue box) was fused to the ATG-deleted open reading frame of EGFP (green box) in the pCS2+ plasmid. The red coloured bar denotes the targeted site recognised by the MO. (F) Quantitative PCR analysis of *mvda* level when *mvda* knockdown through *mvda* morpholino. **, *P* < 0.01.


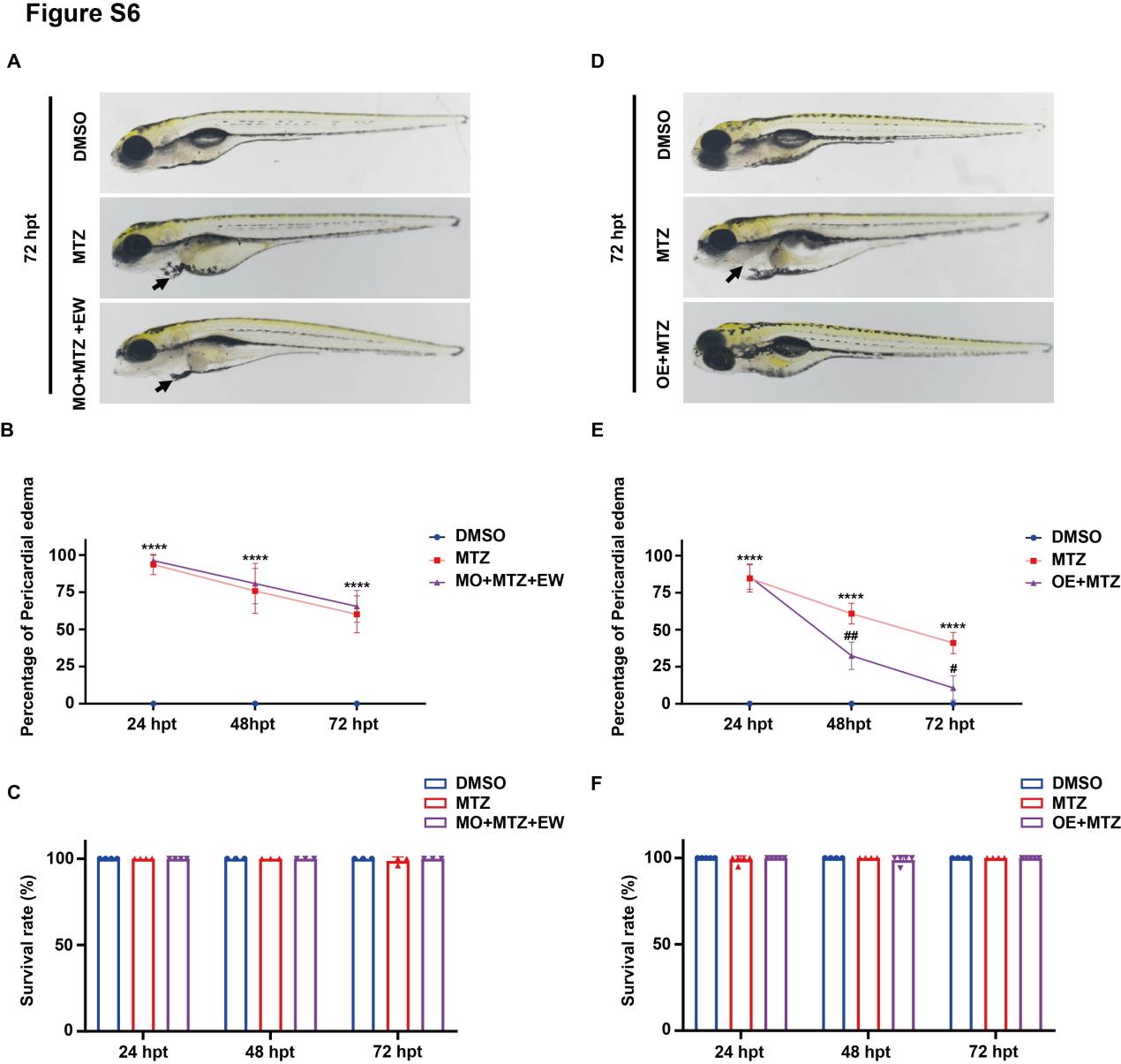


**Figure S6. Morphological changes in *mvda* knocking down and overexpression groups.** Representative morphological images of zebrafish in *mvda* MO group (A) and *mvda* overexpression group (D). The percentage of pericardial edema in *mvda* MO group (B) and *mvda* overexpression group (E). ****, *P* < 0.0001 as compared with DMSO control group. #, *P* < 0.05; ###, *P* < 0.001 as compared with MTZ group. The survival rate in *mvda* MO group (B) and *mvda* overexpression group (E). The black arrow indicate pericardial edema.


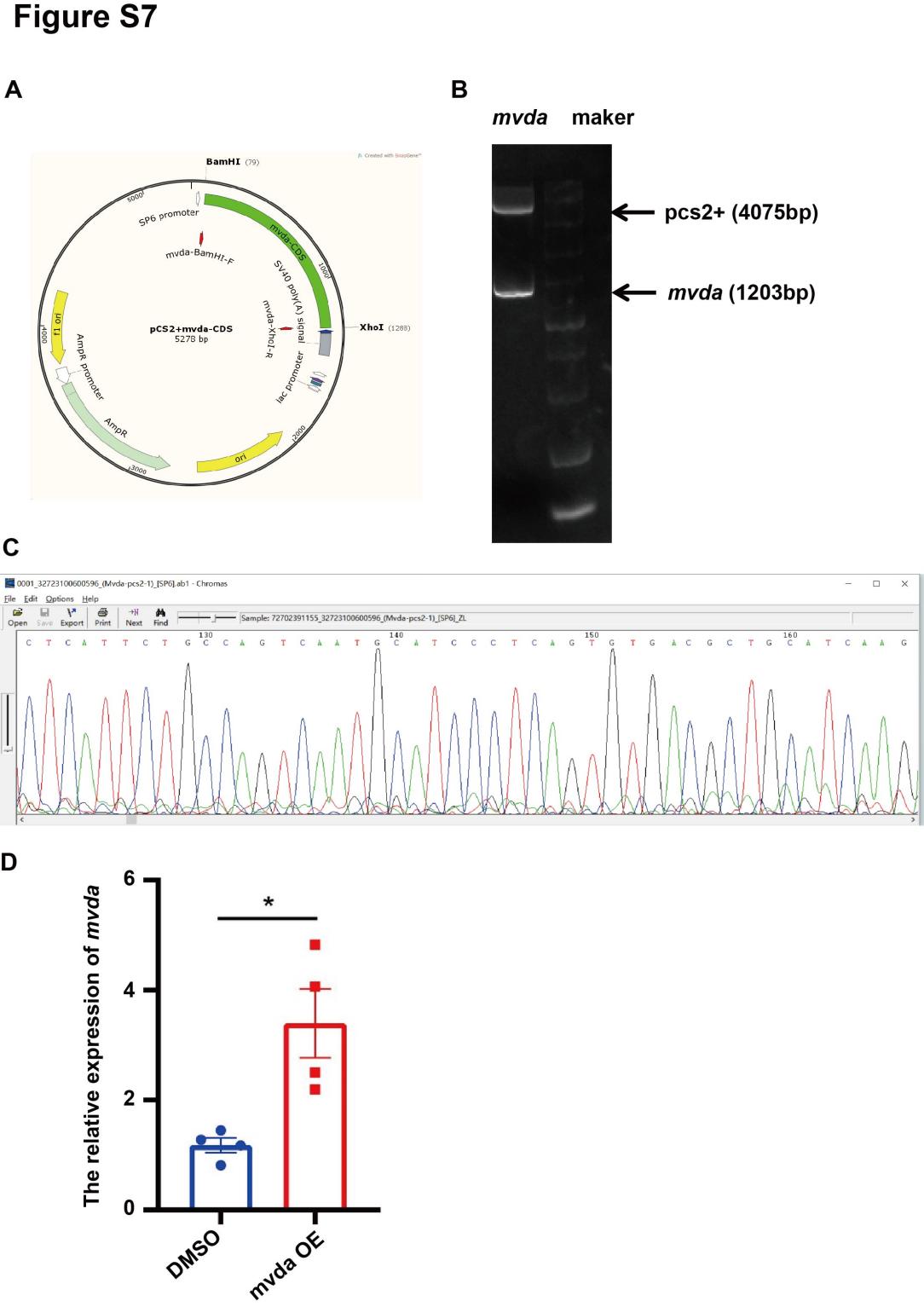


**Figure S7. Construction of *mvda* overexpression plasmid.**

(A) Schematic diagram of *mvda*-pcs2+ plasmid. (B) Agarose gel electrophoresis of *mvda*-pcs2+ plasmid double digested with BamHI and XhoI. (C) Sequencing electropherogram of the *mvda*-pcs2+ plasmid. (D) Quantitative PCR analysis was performed to confirm *mvda* overexpression. **P* < 0.05.


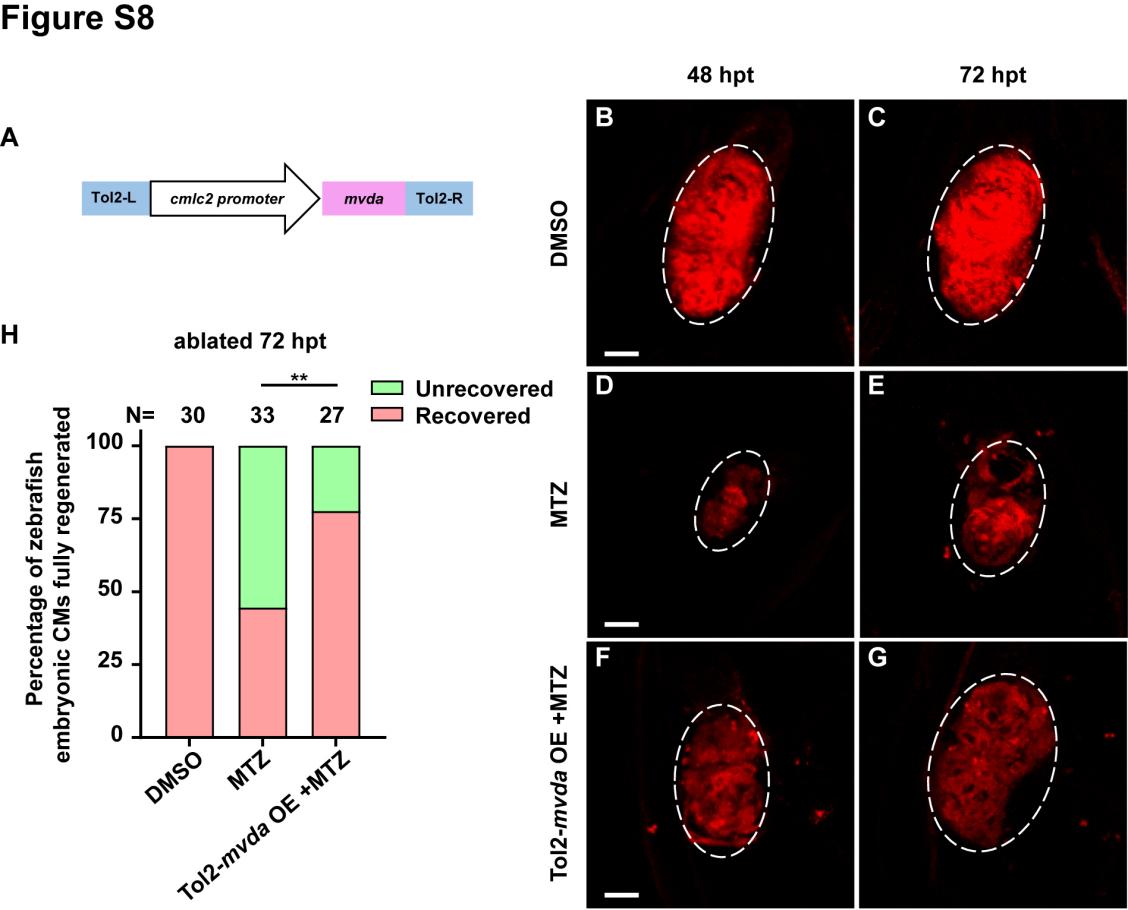


**Figure S8. Cardiomyocyte-specific genetic tools for overexpressing *mvda*.**

(A) Schematic presentation of the Tol2-*cmlc2* promoter-*mvda* plasmid used to overexpression *mvda* in cardiomyocyte. (B-G) Representative images of ventricular morphology of zebrafish in the control (B,C), MTZ (D,E), and cardiomyocyte-specific overexpression group (F, G). Statistical analysis of ventricular regeneration (H). **, *P* < 0.01.

**
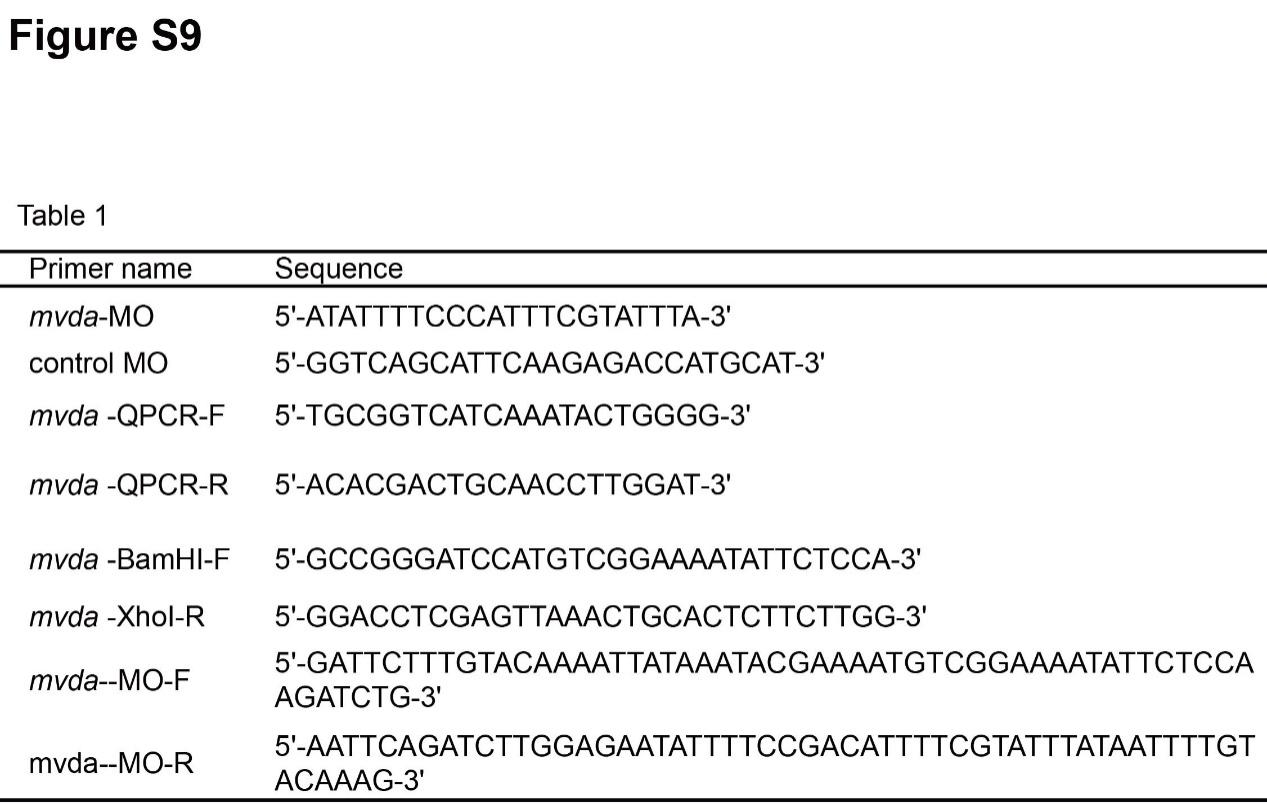
**

**Figure S9. Primer Sequence.**

**Movie S1-3 showed single slide images for the fluorescent image, related to Fig. 2A-C.** The ventricular morphology was showed at 48 hpt (Movie S1), 72 hpt (Movie S2) and 96 hpt (Movie S3) of DMSO group.

**Movie S4-6 showed single slide images for the fluorescent image, related to Fig. 2D-F.** The ventricular morphology was showed at 48 hpt (Movie S4), 72 hpt (Movie S5) and 96 hpt (Movie S6) of MTZ group.

**Movie S7-9 showed single slide images for the fluorescent image, related to Fig. 2G-I.** The ventricular morphology was showed at 48 hpt (Movie S7), 72 hpt (Movie S8) and 96 hpt (Movie S9) of MTZ+EW group.

**Movie S10-12 showed single slide images for the fluorescent image, related to Fig. 2J-L.** The ventricular morphology was showed at 48 hpt (Movie S10), 72 hpt (Movie S11) and 96 hpt (Movie S12) of EW alone group.

**Movie S13-15 showed single slide images for the fluorescent image, related to Fig. 6A.** The ventricular morphology was showed at 48 hpt (Movie S13), 72 hpt (Movie S14) and 96 hpt (Movie S15) of DMSO group in Fig. 6A.

**Movie S16-18 showed single slide images for the fluorescent image, related to Fig. 6A.** The ventricular morphology was showed at 48 hpt (Movie S16), 72 hpt (Movie S17) and 96 hpt (Movie S18) of MTZ group in Fig. 6A.

**Movie S19-21 showed single slide images for the fluorescent image, related to Fig. 6A.** The ventricular morphology was showed at 48 hpt (Movie S19), 72 hpt (Movie S20) and 96 hpt (Movie S21) of MO+MTZ+EW group in Fig. 6A.

**Movie S22-24 showed single slide images for the fluorescent image, related to Fig. 6G.** The ventricular morphology was showed at 48 hpt (Movie S22), 72 hpt (Movie S23) and 96 hpt (Movie S24) of DMSO group in Fig. 6G.

**Movie S25-27 showed single slide images for the fluorescent image, related to Fig. 6G.** The ventricular morphology was showed at 48 hpt (Movie S25), 72 hpt (Movie S26) and 96 hpt (Movie S27) of MTZ group in Fig. 6G.

**Movie S28-30 showed single slide images for the fluorescent image, related to Fig. 6G.** The ventricular morphology was showed at 48 hpt (Movie S28), 72 hpt (Movie S29) and 96 hpt (Movie S30) of OE+MTZ group in Fig. 6G.
